# Assisted migration is plausible for a boreal tree species under climate change: A quantitative and population genetics study of trembling aspen (*Populus tremuloides* Michx.) in western Canada

**DOI:** 10.1002/ece3.9384

**Published:** 2022-10-05

**Authors:** Chen Ding, Jean S. Brouard

**Affiliations:** ^1^ Western Gulf Forest Tree Improvement Program Texas A&M Forest Service, TAMU System College Station Texas USA; ^2^ Isabella Point Forestry Ltd. Salt Spring Island British Columbia Canada

**Keywords:** assisted migration, budbreak, genetic parameters, hardiness, *Populus tremuloides* Michx., suboptimality

## Abstract

A novel method was tested for improving tree breeding strategies that integrate quantitative and population genetics based on range‐wide reciprocal transplant experiments. Five reciprocal common garden tests of *Populus tremuloides* were investigated including 6450 trees across western Canada focusing on adaptation traits and growth. Both genetic parameters and home‐site transplant models were evaluated. We found a genetic trade‐off between growth and early spring leaf flush and late fall senescence. Coefficients of phenotypic variation (*CVp*) of cell lysis (CL), a measure of freezing injury, shrank from 0.28 to 0.10 during acclimation in the fall, and the *CVp* slope versus the freezing temperature was significantly different from zero (*R* ^2^ = 0.33, *p* = .02). There was more between‐population genetic variation in fall phenology than in spring leaf phenology. We suggest that *P. tremuloides* demonstrated a discrepancy between the ecological optimum and the physiological optimum minimum winter temperature. The sub‐optimal growing condition of *P. tremuloides* is potentially caused by the warmer ecological optimum than the physiological optimum. Assisted migration and breeding of fast growers to reforest cooler plantation sites can improve productivity. Transferring the study populations to less than 4°C of extreme minimum temperature appears safe for reforestation aligning with the historical recolonization direction of the species. This is equivalent to a 5–10° latitudinal northward movement. Fall frost hardiness is an effective criterion for family selection in the range tested in this study.

## Graphical abstract

The sub‐optimality of local Populus tremuloides demonstrated discrepancies between the ecological optimum and physiological optimum and the tree species tended to express a colder ecological optimum than the physiological optimum. Transferring the study populations (<‐4°C of extreme minimum temperature, EMT) appears safe for reforestation, equivalent to 5‐10° latitudinal northward movement.

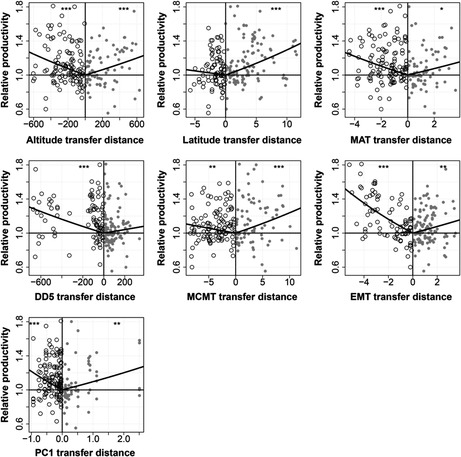

## 1 INTRODUCTION

Global forest ecosystems sequester a large portion of anthropogenic greenhouse gas carbon emissions and mitigate climate change impacts (Blumstein & Hopkins, 2021; Pan et al., 2011). Meanwhile, current and future warming climates may alter the terrestrial ecosystems (Intergovernmental Panel on Climate Change, 1997; Walther et al., 2002) and the ensuing maladaptation will decrease forest productivity and carbon sequestration depending on the interspecific and intraspecific responses of vegetation to climate change (Aitken et al., 2008; Alberto et al., 2013; Anderegg et al., 2016; Hoffmann & Sgrò, 2011; Parmesan, 2006). Among tree species, range shift mortality, and suboptimal growth have been observed in populations of conifers (Barber et al., 2000; Lu et al., 2014; Rehfeldt et al., 2002) and broad‐leaved trees (Anderegg et al., 2016; Vitasse et al., 2009; Worrall et al., 2013).

Adaptive capacity is critical for long‐term productivity with phenotypic acclimation and adaptation of plantation forests as they track environmental cues and seasonality under novel climatic conditions (Nicotra et al., 2010). A warmer climate could trigger an earlier break from winter dormancy (Menzel et al., 2006; Vitasse et al., 2011) or change the senescence phenology leading to maladaptation when the phenology fails to synchronize with the novel climate (Aitken et al., 2008; McKenney et al., 2009). In temperate forests, adaptational lag is less influential in the cold margin populations in terms of height growth (Fréjaville et al., 2020). In boreal forests, there is a strong association between winter coldness and tree species range (Qian et al., 2022). Future reforestation, land reclamation, as well as tree improvement can be informed by the habitat or range shifts as well as the correlated selection and breeding responses among growth, phenology, and physiological traits (Chuine, 2010; Ding, 2015; Etterson et al., 2020; Otis Prud'homme et al., 2018).

Previous studies demonstrated the suboptimal growth condition of multiple commercial tree species that were widely distributed in North America (Lu et al., 2014; Rehfeldt et al., 2018; Schreiber, Ding, et al., 2013). According to the “Namkoong's non‐optimality concept”, trees tend to occur in a cooler range than the optimal climate (Namkoong, 1969). In *Pinus contorta* var. latifolia, for example, the ecological optimum does not align with the physiological optimum climatic condition, where winter cold climate (negative degree‐days) is a key driver to determine the population growth potential (Rehfeldt et al., 2018). Some angiosperm trees may already be maladapted to the current climate due to discernible adaptational lags, for example, cooler optimum growth temperatures than the current (Browne et al., 2019). Assisted gene flow through reforestation needs to be evaluated for mitigating this maladaptation impact (Browne et al., 2019).

Long‐term and short‐term common garden experiment data on phenology and cold hardiness can determine the drivers of adaptation, as well as the genotype‐environment effects on trees (Isabel et al., 2020; White et al., 2007). With long‐term field common gardens the genetic parameters are estimable to inform the population structures and within‐population genetic variations. Studying provenance effects and genetic variance and covariance of phenology traits can help tree breeders and foresters in reforestation and plantation in the following ways: (1) assisting optimal genotype or family to migrate to sites without maladaptation (Gray & Hamann, 2011; Joyce & Rehfeldt, 2017; Koralewski et al., 2015; Montwé et al., 2018; Schreiber, Ding, et al., 2013); or (2) exploiting the current genetic variance and covariance of adaptation and productivity of elite families for breeding programs (Clair et al., 2010; Kanaga et al., 2008; Pliura et al., 2014). Other adaptation strategies include mixed species in plantation stands, mixed families, and provenance groups in deployment, as well as increasing genetic diversity of reforestation with multiple ages of stands (Williams & Dumroese, 2013).

Bud break and growth cessation determine the growing season length (Cooke et al., 2012; Rohde et al., 2011; Vitasse et al., 2009). Substantial genetic control and clinal variations of multiple adaptive traits were reported among the *Populus* species in North America and Europe (McKown et al., 2014; Olson et al., 2013). Complex physiological mechanisms of these adaptive traits are trait‐specific and are driven by different genetic architectures of the traits and express various genetic clines (Howe et al., 2003). In *Populus tremula*, the genetic variation of bud set and growth cessation showed a latitudinal cline and demonstrated the trade‐off between cold hardiness and growth increment (Hall et al., 2007; Luquez et al., 2008). In *P. trichocarpa*, substantial genetic variation and genetic correlation were found among the phenological transition and growth traits and the potential adaptability was expected to be expressed during climate change (Richards et al., 2020).

Frost risks in the spring and fall are critical periods associated with long‐term survival and productivity. The complexity of the cold hardiness trait suggests that many underlining physiological and biochemical processes are involved and regulated by genetic and epigenetic factors (Wisniewski et al., 2018). Artificial freezing tests that mimic the natural frost damage to tissues can quantify the physiological response at the individual and family levels. Cold adaptation remains a critical criterion for assisted migration of trees (Montwé et al., 2018). Avoiding frosts results in late spring and early fall phenology (Schreiber, Ding, et al., 2013). Tissue damage due to extreme cold conditions is a limiting factor for tree survival in boreal plantation sites during winter conditions (Howe et al., 2003; Schreiber, Hamann, et al., 2013). In a tree breeding and reforestation program, a mismatching of acclimation timing is likely to raise frost risks of planted trees, however extremely low temperatures cause less lethal damage after acclimation (Hall et al., 2007).

Higher genetic variations of spring phenology in tree species were reported compared to that of fall and cold adaptive traits (Howe et al., 2003). The coefficient of additive genetic variation (*CV*_*A*_) is the additive genetic variance (*V*_*A*_) per unit of the trait mean that was subjected to less influence of environment variances compared to the heritability (Csilléry et al., 2020; Garcia‐Gonzalez et al., 2012; Miller & Penke, 2007). *CV*_*A*_ refers to the ability of natural and artificial selection and it measures the “evolvability” or selection potential (Lynch & Walsh, 1998). This parameter can be as important as heritability when evaluating trait genetic variation, particularly in low heritability traits. Population differentiation of quantitative traits can be represented using the index *Q*_*st*_ based on common garden observations (Spitze, 1993). *Q*_*st*_ measures the genetic differentiation among populations for quantitative traits such as growth and phenology; *F*_*st*_ measures the population divergence based on neutral genetic marker variances and the selection among the natural populations can be inferred from the comparison of *Q*_*st*_ and *F*_*st*_, to reveal the evolutionary and ecological dynamics and insights for multiple fitness, growth, adaptive, and morphological traits (Csilléry et al., 2020; Yang et al., 1996).

*Populus tremuloides* Michx. is one of the most wide‐distributed tree species in North America, and an early successional species that provides benefits for ecosystem service as well as social‐economical values (Perala, 1990). Currently, the species range may shift under climate change by losing historical habitat in the southern range in the United States and the parkland in Alberta, Canada (Worrall et al., 2013). The mortality and yield losses are due to the cumulative climate change impacts. This may jeopardize the interest of the forestry sectors and local communities (Morelli & Carr, 2011). Evolutionary adaptation of the species is linked with the repeated recolonization from multiple refugia in the United States with those populations expanding to the current species range under a warming climate trend (Ding et al., 2017). *P. tremuloides* demonstrates discernible cold tolerance in the light of the climates they adapted to after the Last Glacier Maximum (Brouard, 2004; Ding et al., 2017; Girardin et al., 2022). The cooler ecological optimum of the species will not match current and future warmer climatic conditions, which could lead to maladaptation and potential decline of local populations. To cope with potential risks of maladaptation, assisted migration was proposed as a means to maintain productivity in commercial plantations (Gray et al., 2011). In *P. tremuloides*, the suboptimality was reported as a trade‐off between growth and avoidance of frost risks in fall. Early fall senescence reduces the risk of frost injury while reducing the growing season length (Schreiber, Ding, et al., 2013); However, different spring and fall adaptation behaviors at the genetic level and the relation to the climatic gradient are not clearly understood in the context of the range‐wide reciprocal transplant experiments.

In this study, we investigated the relationship between local adaptation and genetic parameters of the adaptive traits and growth trait of *P. tremuloides* based on five reciprocal common garden experiments with 43 half‐sib families from six biogeoclimatic regions (Schreiber, Ding, et al., 2013). We hypothesized that spring and fall phenology have diverged selection pressures and the distinctions of these adaptation characteristics contribute to the current suboptimality growth of *P. tremuloides* covering the Canadian range of the species. The responses of current experiments for height growth, productivity, and adaptive traits inform future mitigation and reforestation operations. Our main aims were (1) evaluating the quantitative genetic parameters of growth and adaptive traits, such as genetic correlation (*r*_*G*_), coefficient of additive genetic variation (*CV*_*A*_), narrow‐sense heritability (*h* ^*2*^ *)*, etc.; (2) investigating the genetic architecture of frost hardiness of *P. tremuloides* in the context of acclimation; (3) comparing the adaptation mechanisms of the spring and fall phenology under seasonal frosts given the suboptimal growth trend (i.e., deviations between the ecological and physiological optima) under climatic gradients. We demonstrate the reasons for adopting assisted migration to counter this maladaptation, especially in reforestation.

## 2 MATERIALS AND METHODS

### 2.1 Study area and plant materials

**FIGURE 1:**
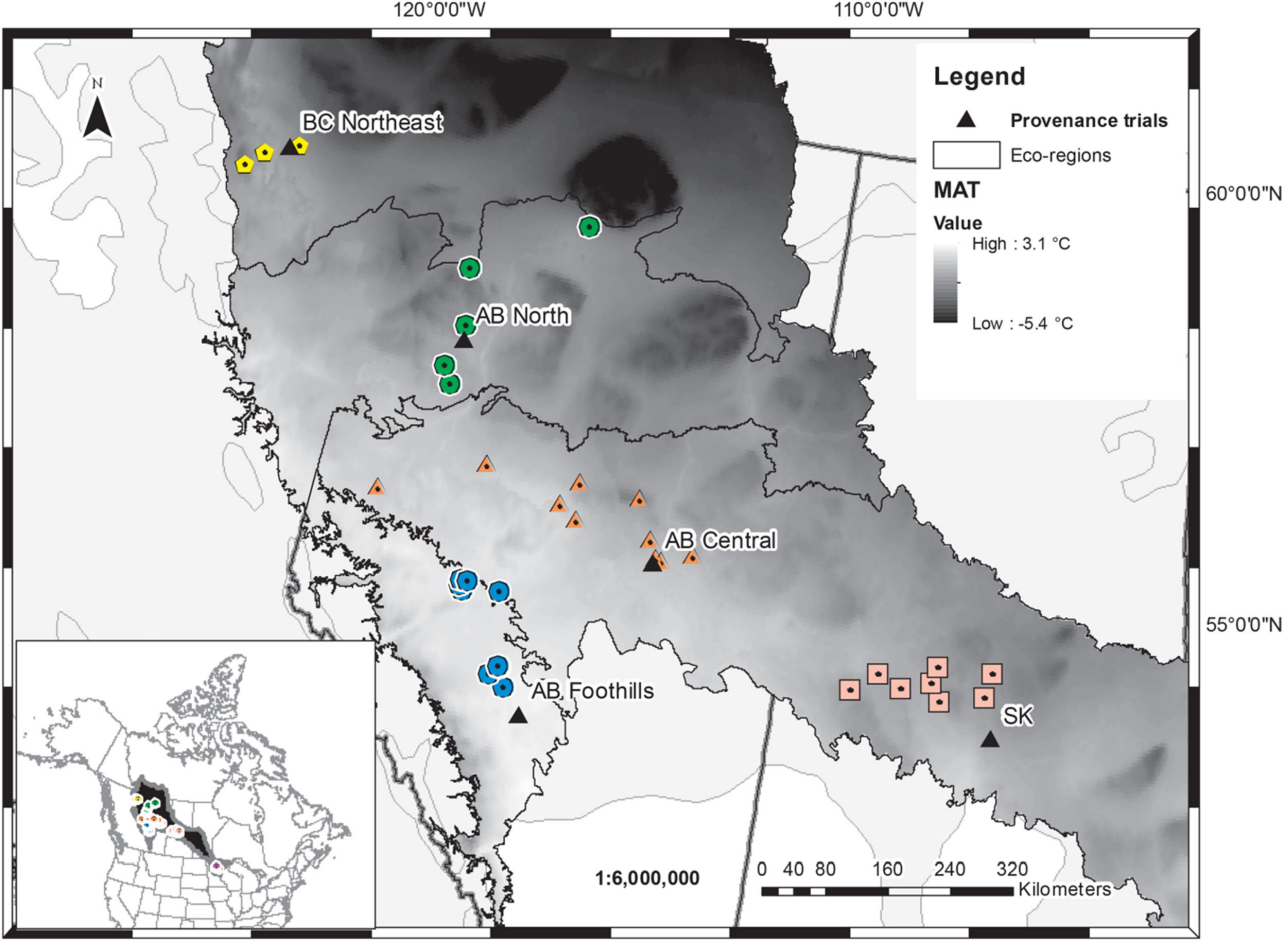
Seed collection locations (43 families), five common garden trial sites, and mean annual temperature (MAT, °C). MAT, 1961–1990 climate norms from ClimateWNA (2015) across the range of western Canada. The six provenance groups were British Columbia (BC) Northwest, Alberta (AB) North, AB Central, AB Foothills, Saskatchewan (SK), and Minnesota in the United States. Each common garden was established in a Canadian provenance group as marked by the triangles. Adaptive traits were measured only at trial Alberta Central (#60). The provenances were marked by different icons in eco‐regions that indicate five major Canadian populations on the main frame of the map while the five Minnesota families were marked also in the smaller data frame on the left. The light gray area shows the natural range of *Populus tremuloides*, and the ecoregions were within the habitat. Minnesota populations were not shown in the temperature map due to the limitation of the ClimateWNA data coverage at a longitude of 100° W.

The raw growth datasets reanalyzed here are documented in a previous study (Schreiber, Ding, et al., 2013). The study area covers western Canada (i.e., northern British Columbia, Alberta, Saskatchewan) and Minnesota State, USA. There were 43 single tree collections (putative half‐sib families) sampled across the study area and they were clustered into six ecoregions (termed provenance groups). Thousands of germinated seeds were produced from the sampled trees. There were five reciprocal common gardens established in the five Canadian ecoregions to test the genetic differences in western Canada. Five Canadian provenance groups and one Minnesota group were delineated among the 43 half‐sib families based on the ecoregions and biogeoclimatic conditions (Figure 1) thus these 43 families are nested within six ecoregions or provenances. To capture a larger landscape of genetic variation, the experiment also sampled an additional five half‐sib families from the boreal shield ecoregion in the Minnesota State, USA (MN), without trial establishment due to administration, budget, and logistic constraints. Here, the local and transplants were compared in the trials in British Columbia Northwest (BC, Trial #70), Alberta North (Abn, Trial #10), Saskatchewan (SK, Trial #90), AB Central (Abc, Trial #60), AB Foothills (Abf, Trial #33). A detailed description of the five reciprocal common garden trials is given in Table 1, Table S1 including the experimental design, the number, and origins of the populations, locations (also in Figure 1), and the year of trial establishment. All test sites were established in spring 1998 from over‐winter dormant stock and planted as randomized complete blocks with 43 family treatments, in six replicates of five‐tree‐row plots. To avoid edge effects, each trial was surrounded by two rows of border trees.

**TABLE 1:** Geographic location and climatic variables of 43 *Populus tremuloides* populations in western Canada and Minnesota state, and five common garden test sites of provenance trials

Populations groups	Provenance #	Latitude (N)	Longitude (W)	Elevation (m)	MAT (°C)	MWMT (°C)	MCMT (°C)	MAP (mm)	MSP (mm)	eFFP	EMT (°C)
*British Columbia Northeast* (BC) *AB North* (nAB)	759*	58°12′	123°20′	1177	−0.7	12.5	−12.8	722	496	242	−46.2
	760*	58°36′	122°20′	335	−0.5	16.5	−19.5	443	297	248	−49.4
	793	58°24	123°0′	511	0.2	15.5	−15.6	507	345	248	−47.7
	752	56°25′	117°46′	739	0.4	15.1	−18.0	433	274	247	−44.7
	753	57°7′	117°44′	606	−0.2	15.5	−20.3	431	272	245	−44.7
	754	56°37′	117°59′	709	0.2	15.3	−18.7	437	276	248	−44.8
	755	57°47′	117°58′	459	−0.5	15.9	−21.8	430	268	247	−43.8
	756	58°34′	115°38′	343	−1.0	16.9	−23.0	391	240	253	−45.9
*Alberta Central* (cAB)	757	55°11′	114°37′	726	1.4	15.2	−15.0	545	382	253	−45.9
	758	55°20′	115°1′	646	1.5	15.5	−15.1	530	370	255	−45.3
	761	55°36′	116°40′	632	1.3	15.7	−16.6	485	319	251	−45.9
	762	55°36′	116°40′	632	1.3	15.7	−16.6	485	319	251	−45.9
	763	55°36′	116°40′	632	1.3	15.7	−16.6	485	319	251	−45.9
	764	55°38′	114°41′	709	0.6	15.1	−17.2	537	366	251	−45.5
	777	54°56′	112°52′	546	1.2	16.2	−17.2	462	319	251	−46.1
	778	55°8′	113°1′	601	0.8	15.8	−17.6	475	327	249	−46.3
	779	55°4′	112°7′	624	0.7	15.7	−17.3	456	318	249	−46.3
	775*	55°36′	113°25′	762	−0.7	14.8	−20.4	507	342	242	−45.8
	776*	54°56′	112°44′	545	1.2	16.2	−17.1	458	317	251	−46.1
	751	55°2′	118°44′	649	1.6	15.7	−14.9	476	306	250	−47.2
*Alberta Foothills* (ABf)	765	54°11′	115°47′	731	2.1	15.7	−13.4	563	383	251	−46.1
	766	54°6′	116°30′	1018	2.4	14.6	−11.0	624	442	253	−43.3
	767	54°8′	116°35′	868	2.5	15.2	−11.8	606	432	253	−44.0
	768	54°13′	116°26′	803	2.2	15.3	−12.9	591	419	253	−45.1
	769	54°13′	116°26′	803	2.2	15.3	−12.9	591	419	253	−45.1
	770	54°13′	116°35′	914	2.4	15.1	−12.0	607	433	254	−44.4
	771	53°12′	115°36′	939	2.4	15.0	−12.0	599	433	253	−44.9
	772	53°19′	115°28′	939	2.3	15.1	−12.1	592	427	252	−45.1
	773	53°18′	115°26′	927	2.4	15.1	−12.1	590	426	252	−45.1
	774	53°5′	115°16′	912	2.5	15.4	−12.1	586	424	254	−44.8
*Saskatchewan* (SK)	780	54°12′	105°42′	490	−0.3	16.9	−20.5	470	306	250	−46.1
	781	54°12′	106°48′	513	0.0	16.9	−20.1	453	302	250	−45.7
	782	54°0′	106°54′	519	0.2	17.0	−19.7	446	296	251	−45.5
	783	53°54′	105°48′	517	−0.2	16.9	−20.4	457	300	250	−45.7
	784	53°48′	106°42′	583	0.4	16.6	−18.6	437	291	252	−45.7
	785	54°2′	108°0′	530	0.5	17.0	−19.2	443	303	249	−45.5
	786	53°48′	108°30′	710	−0.2	15.9	−19.3	457	313	245	−46.2
	787	53°54′	107°30′	570	0.3	16.7	−19.4	440	297	250	−45.2
*Minnesota* (MN)	788	47°0′	93°0′	384	4.0	19.5	−14.6	700	457	262	−47.1
	790	47°36′	93°24′	424	3.1	19.0	−16.1	678	451	257	−47.9
	792	47°12′	93°24′	395	4.0	19.8	−15.1	696	455	262	−47.3
	789*	47°12′	93°48′	405	3.9	19.8	−15.1	691	452	261	−47.3
	791*	47°30′	93°36′	433	3.0	19.0	−16.3	682	452	257	−48.0

Test sites	Location	Latitude (N)	Longitude (W)	Elevation	MAT	MWMT	MCMT	MAP	MSP	eFFP	EMT
*nAB, #10*	Manning	56°58′	117°44′	570	0.2	15.8	−19.8	419	266	247	−44.5
*ABf, #33*	Medicine Lake	52°44′	114°47′	973	2.6	15.3	−11.7	558	396	255	−44.6
*cAB* ^*l*^_._ *#60*	Athabasca	54° 53′	112° 52′	570	1.2	16.0	−17.0	469	323	251	−46.1
*BC, #70*	Fort Nelson	58°32′	122°20′	335	−0.4	15.9	−18.0	453	305	248	−49.0
*SK, #90*	Prince Albert	53°20′	105°36′	480	0.3	17.3	−20.2	431	284	251	−45.3

*Note*: The provenances with asterisks were sampled for assessment of frost hardiness (2011), tree physiological parameters (2010), and tree height (2009). MAT, mean annual temperature (°C); MWMT, mean warmest month temperature (°C); MCMT mean coldest month temperature (°C); MAP, mean annual precipitation (mm); MSP, mean May‐to‐September precipitation (mm); eFFP, the Julian date on which the frost‐free period ends (DoY); EMT, extreme minimum temperature over 30 years (°C).

^*^ Provenances tested in the cold hardiness test.

Tree height was measured for 6450 individual trees after nine growing seasons in five reciprocal common garden trials in the autumn of 2008, where approximately 1290 trees were measured in each common garden excluding the border and filling trees. Phenological measurements, for example, the timing of bud break and leaf senescence, were recorded on 1290 trees at the central Alberta common garden trial (#60).

### 2.2 Phenology data

Raw bud break scores were obtained from a previous study (Li et al., 2010), and leaf senescence scores were taken on seven consecutive dates: September14, 18, 21, 23, 25, 28, and October 2, 2010, based on a previous study (Schreiber, Ding, et al., 2013). Scoring was based on a lately modified eight‐level senescence scale according to Fracheboud et al. (2009), and our newly estimated scores were analyzed from the beginning of the phenology events to their end stages which were represented by the highest score. The day of year (DoY) was inferred by the linear regression from the bracketing dates and scores. To estimate the genetic variances among families, we chose the critical scores representing consecutive stages of the spring and fall phenological traits (Table S7). Here, we evaluated the continuous phenology processes with the raw data (spring growth initiation and fall acclimation processes) and included transition phenological stages ranging from the beginning to the end of the score series (e.g., 1.5, 2.5, 3.5, 4.5, etc.).

### 2.3 Climate data and frost risk assessment

Climate PP and ClimateWNA software packages were employed to obtain precise spatial bioclimatic variables (Mbogga et al., 2009; Wang et al., 2012) both at the provenance origins and at common garden trials. We used the normal climate variables (1961–1990) as the long‐term baseline climate for the study area and provenance groups (Table 1).

To study the correlation between phenology and climatic conditions, Trial #60 was selected in central Alberta, in which individual tree phenology and height were both measured. The daily weather data were retrieved from the *National Climate Data and Information Archive* (Environment Canada; http://www.climate.weatheroffice.gc.ca), and the daily weather dataset covered the 1961–1990 normal period and the actual growing period of trees (1998–2010). The maximum and the minimum temperatures were between ±20°C, with annual precipitation around 474 mm, as a typical continental climate. These weather station data were applied to evaluate the degree days of the spring growing period (heat‐sum, daily maximum temperature > 0°C). Here, the heat‐sum of bud break was calculated with 0°C as the base temperature.

### 2.4 LT50 prediction and frost risk assessment

The frost hardiness of northern British Columbia (BC), central Alberta (cAB), and Minnesota (MN) provenance groups were measured at the central Alberta trial (#60). We chose the three populations to represent a northwest to southeast transect of study populations aligning with the species range in order to reveal the full range of the intraspecific genetic variation of the hardiness trait. Lethal damage temperatures of 50% (LT50) and 25% cell lysis (LT25) were quantified and predicted. The experimental design, procedure, raw data, and cell lysis (CL) calculation method are documented by Schreiber, Ding, et al. (2013).

The LT50 was measured using the electrolyte leakage method with the artificial freezing treatment, which simulated the processes of frost damage to the plasma membranes of twig tissues. Tree twigs were sampled at the cAB test site from two families in each of the three provenance groups (Minnesota MN, central Alberta cAB, and northeast British Columbia nBC) representing the full continental transect of the population range of the study area. Eight trees were randomly chosen per family and current year twigs per freezing treatment were collected. The collection was repeated three consecutive times with 20–31 days' intervals from 22 August to 10 Oct 2011. All twigs from 48 trees per collection date were cut into 5 cm pieces and placed in 30 ml high‐density polyethylene bottles and were added 5 ml of deionized water before freezing treatments. The freezing treatments during the three sampled times were listed in the Table S8. One twig was used for each freezing temperature. A programmable freezer (Model 85–3.1A, Scientemp Corp.) cooled the samples at a rate of 5°C per hour, holding the target temperature for 1 h before re‐warming to 8°C. Each segment was subsequently cut into 5 mm pieces, topped up with 20 ml deionized water, stored for 20–24 h at 8°C, and manually shaken three times during the storage. Each temperature treatment was applied to 48 sampled individual trees (i.e., 3 populations × 2 families per population × 8 trees/family). The amount of electrolyte leakage was measured at room temperature (approximately 20°C) using a conductivity meter (Oakton Acorn CON 6 Meter, Oakton Instruments). Here, we also predicted LT50 and LT25 cell lysis temperature (°C) given the sampling DoY for each half‐sib families; and then the genetic parameters of cell lysis were estimated. A relative electrolyte leakage ratio (*REL*) was determined by the quotient of the first conductivity measurement after freezing (*C*_*1*_) against the second measurement of the heat‐killed samples (*C*_*2*_) (Morin et al., 2007). The index of cell lysis (%) described the degree of hardiness (Morin et al., 2007):

(1) $$L=\frac{\mathrm{REL}-\bar{{\mathrm{REL}}_{C}}}{100-\bar{{\mathrm{REL}}_{C}}}$$

where *REL* = $\frac{C_{1}}{C_{2}}$, $\bar{{\mathrm{REL}}_{C}}$ was the mean value of the control for each family. The temperature causing LT50 was regarded as the lethal temperature that caused half‐cell damage; here LTs were estimated with quadratic functions explained in the supplementary (Method S11).

The probability of daily freeze–thaw events in winter was calculated from 1960 to 2010 based on the historical daily weather data at the central Alberta site (Environment Canada, Station 306,032) in Figure 3. The weather data were shown and a warmer and dryer climate trend was projected (Figure S2). The freeze–thaw events during the winter were calculated as the difference between max daily temperature (TMAX) and min daily temperature (TMIN) when the max daily temperature was equal to or greater than 5°C and TMIN was equal to or less than −5°C. The probability (0%–100%) was calculated based on the historical daily data from 1960 to 2010 as 100%*(the counts of freeze–thaw events by DoY/number of years of records of that DoY).

### 2.5 Quantitative genetic parameters

Additive genetic and phenotypic variances were calculated with the SAS 9.3 PROC MIXED (SAS Institute). In order to estimate the within‐population variance by each population, we grouped each provenance groups of half‐sib families resulting in the following family effect model:

(2) $$y_{\text{inkm}}=\mu +B_{i}+P_{n}+F_{\mathrm{kn}}+{\mathrm{BF}}_{\mathrm{ink}}+e_{\text{inkm}}$$

where $y_{\text{inkm}}$ was the trait observation of the *m‐*th tree in the *k*‐th half‐sib family of the *i*‐th block from the *n*‐th population; μ was the grand mean; $B_{i}$ was the fixed effect of *i*‐th block; $P_{n}$ was the random effect of *n*‐th population; and $F_{\mathrm{nk}}$ was the random effect of *k*‐th half‐sib family within *n*‐th population; $e_{\text{inkm}}$ was the random residue of *m*‐th each tree. The REPEATED statement was used to estimate the within‐plot residual variance and there were ~43 plots nested in each rep. $P_{n}$, $F_{\mathrm{nk}}$, ${\mathrm{BF}}_{\mathrm{ink}}$, and $e_{\text{inkm}}$ were assumed to follow normal independent identical distributions $N_{\mathrm{iid}}\left(0,\sigma_{\mathrm{pop}}^{2}\right)$, $N_{\mathrm{iid}}\left(0,\sigma_{f}^{2}\right)$, $N_{\mathrm{iid}}\left(0,\sigma_{\mathrm{bf}}^{2}\right)$,$N_{\mathrm{iid}}\left(0,\sigma_{e}^{2}\right)$, respectively.

Additive genetic variance (${\hat{\sigma }}_{A}^{2}$) equaled to four times of the half‐sib family variance component (*V*_*f*_); the phenotypic variance (${\hat{\sigma }}_{P}^{2}$) was ($\hat{\sigma_{f}^{2}}+\hat{\sigma_{E}^{2}}$) (Falconer & Mackay, 1996; Isik, 2012). The narrow‐sense heritability (${\hat{h}}^{2}$) was calculated with the following function:

(3) $$\hat{h^{2}}=\frac{\hat{\sigma_{A}^{2}}}{\hat{\sigma_{f}^{2}}+\hat{\sigma_{E}^{2}}}=\frac{4V_{f}}{V_{f}+V_{E}}\;$$

The ${\hat{Q}}_{\mathrm{st}}$ index was calculated as

(4) $${\hat{Q}}_{\mathrm{st}}=\frac{{\hat{\sigma }}_{B}^{2}}{{\hat{\sigma }}_{B}^{2}+2{\hat{\sigma }}_{A}^{2}}=\frac{V_{\mathrm{pop}}}{V_{\mathrm{pop}}+8V_{f}}$$

where the ${\hat{\sigma }}_{B}^{2}$ was the between‐population variance component (V_pop_); ${\hat{\sigma }}_{A}^{2}$ was four times of V_f;_ V_e_ was the random residual. Delta method was applied to calculate the standard error of ${\hat{\sigma }}_{A}^{2}$, ${\hat{\sigma }}_{P}^{2}$, ${\hat{Q}}_{\mathrm{st}}$ and ${\hat{h}}^{2}$. Coefficients of variation (*CV*) were estimated as.

(5) $${\mathrm{CV}}_{A}=\frac{\sqrt{{{\hat{\sigma }}_{A}}^{2}}}{m},{\mathrm{CV}}_{P}=\frac{\sqrt{{{\hat{\sigma }}_{P}}^{2}}}{m},{\mathrm{CV}}_{E}=\frac{\sqrt{{{\hat{\sigma }}_{e}}^{2}}}{m}$$

where the *CV* of additive genetic variance was (*CV*_*A*_); coefficients of phenotypic variance was (*CV*_*P*_); coefficients of residual was (*CV*_*E*_); m was the trait mean.

For the bivariate analyses, the linear model was as follows,

(6) $$\left[\begin{array}{c} y_{i}\\ y_{j} \end{array}\right]=\mathrm{x\beta}+{\mathrm{Za}}_{\left(t\right)}+e$$

where yi denoted the phenotypic value of trait i; β was fixed effect of block, population, and grand mean as described in the previous model (2); a_(t)_ was the random family effect within trait $a\left(t\right)\sim N\left(0,f⊗V_{f}\right),$where the f was the identity index matrix of half‐sib families and V_f_ was the half‐sib family variance ($1/4{\hat{\sigma }}_{G_{i}}$); and ewas the residual error, with $e\sim N\left(0,I_{e}⊗V_{R}\right)$. The bivariate models were analyzed with the ASREML‐R v3.0 package (Butler et al., 2007).

The additive genetic correlation (${\hat{r}}_{\mathrm{Gij}}$) was as the following

(7) $${\hat{r}}_{G_{\mathrm{ij}}}=\frac{{\hat{\sigma }}_{G_{\mathrm{ij}}}}{{\hat{\sigma }}_{G_{i}}{\hat{\sigma }}_{G_{j}}}$$

where ${\hat{\sigma }}_{G_{\mathrm{ij}}}$ denotes the estimate of genotypic covariance between traits i and j. ${\hat{\sigma }}_{G_{i}}$ was the estimated genotypic variance of trait j, which was the family covariance parameter. To adjust the differences due to different units and scales between growth and adaptive traits, the natural logarithm transformed values of traits were employed.

For the genetic analysis of CL, an individual tree model was used in a reduced function due to the sample size change from ~1290 trees/trial to 48 trees per freezing test temperature, as follows:

(8) $$y_{\mathrm{nm}}=\mu +P_{n}+A_{m}+e_{\mathrm{mn}}$$

where $A_{m}$ is the random additive genetic effect *m*‐th individual tree; *P*_*n*_ is the fixed effect of population n; *e*_*mn*_ is the random residual of *m*‐th tree in *n*‐th population at a treatment temperature. The upper and lower confidence intervals (97.5% and 2.5%) of the genetic parameters were calculated with the Jack Knife method (Wu, 1986), when the sample size was not sufficiently large compared to the full field test data (*n* = 48). To facilitate the convergence of the linear models, a constant of 0.25 was added to each CL observation of the individual tree. We resampled 48 times from the original data with 48 deletions of individuals in total, so there were 48 Jackknife replicates constructed. We estimated five freezing temperatures*three sampling dates*48 Jackknife replications of 48 individuals were used for estimating each genetic parameter per treatment level which reduced standard errors of genetic parameters. The linear regression between genetic parameters and the geographic and climatic variables of provenance groups or DoY were constructed with the *lm()* function in the R program.

### 2.6 Home‐site models

Home‐site model was built based on the niche breadth theory with a regression approach (Ishizuka & Goto, 2012; Rehfeldt et al., 2002). First, the productivity of each family was estimated by multiplying the average tree height (m) of the family with its survival (%) at each site and relative productivity of the population was calculated, that is, ${\mathrm{PD}}_{\mathrm{ij}}$ (Ishizuka & Goto, 2012). Thus, ${\text{Height}}_{\mathrm{ij}}$ is the tree height of *j*‐th family at *i* th trial site; ${\mathrm{Sur}}_{\mathrm{ij}}$ denotes the survival of *j* ‐th family at *i*‐ th site; ${\mathrm{PD}}_{\mathrm{ij}}$ is the productivity of *j*‐ th family at *i* ‐th site, where ${\mathrm{PD}}_{\mathrm{ij}}={\text{Height}}_{\mathrm{ij}}\times {\mathrm{Sur}}_{\mathrm{ij}}$. The local productivity (${\mathrm{PD}}_{\text{local}}$) is defined as following: at site #70, the average *PD* of the provenance group BC; at site #10, the average *PD* of the provenance group AB North; at site #33, the provenance group AB Foothills; at site #60, AB Central provenance groups; and at site #90, SK group. No trial data of MN provenance group are available. Relative ${\mathrm{PD}}_{\mathrm{ij}}$ was the quotient of ${\mathrm{PD}}_{\mathrm{ij}}$ and related local productivity (${\mathrm{PD}}_{\text{local}}$). A relative height and relative survival were also fitted as the dependent variables, which were not typical productivity and survival measured directly from the trails but the relative performance contrast against the local seed sources after migration. The distances (dist) of environmental variables (latitude, elevation, MAT, and DD_5) were calculated as the differences between the site condition and the seed origin. Thus, the relationship between the relative productivity and migration distances (dist) was constructed as follows:

(9) $$\ln\left(\frac{{\mathrm{PD}}_{\mathrm{ij}}}{{\mathrm{PD}}_{\text{local}}}\right)={\text{dist}}_{\mathrm{ij}}\times b_{1Z}+b_{0Z}+e$$

where $b_{0Z}$ and $b_{1Z}$ were the coefficients of the function, and *e* was the random residual. Here z denoted the transplanting direction. We estimated $b_{0Z}$ and $b_{0Z}$ with two scenarios: upward (dist >0) direction, and downward (dist <0). For example, when the MAT distance was more than zero, the prediction was for an increasing temperature scenario. Furthermore, we set the intercept to zero when dist = 0 and the dependent variable $\frac{{\mathrm{PD}}_{\mathrm{ij}}}{{\mathrm{PD}}_{\text{local}}}=1$. To summarize the climatic effects, Principal component analysis (PCA) was used to produce the principal component 1 (PC1) as a combined climatic distance variable with the R function *pcomp()*. PC1 indicated heat and growing degree conditions, which accounted for 57% of the total variance. To evaluate the fitting of models, *F‐*test of linear regression and adjusted R^2^ were calculated (Table S6).

## 3 RESULTS

### 3.1 Genetic and phenotypic correlations

Moderate to high additive genetic correlations were found between height and leaf senescence (*r*_*G*_ = 0.5–0.9) that absolute magnitude was more than twice the SE, as well as the height versus growing season length (*r*_*G*_ = 0.5 ± 0.1, Table 2). The spring phenology was negatively associated with height (*r*_*G*_ = −0.2) and bud break was linked to growing season length (*r*_*G*_ = −0.7~−0.9). Spring and fall phenology were not correlated, except bud break of score one versus leaf coloration (*r*_*G*_ = 0.7).

**TABLE 2:** Additive genetic correlations and the standard error (the upper diagonal) between phenology traits and height measured at Athabasca, AB, for *Populus tremuloides*

	Spring			Fall				Duration		
Traits	BUD1	BUD3	LEAF	SEN1	SEN3	LCOR5	LABS	SENDU	LEAFDU	GL
HT	−0.17 (0.09)	−0.24 (0.06)	−0.23 (0.06)	0.51 (0.10)	0.73 (0.09)	0.84 (0.06)	0.82 (0.08)	0.14 (0.12)	0.07 (0.11)	0.53 (0.07)
Spring										
BUD1		0.97 (0.03)	0.87 (0.05)	0.06 (0.07)	0.14 (0.08)	0.68 (0.14)	−0.14 (0.17)	−0.24 (0.40)	−0.57 (ns)	−0.88 (0.06)
BUD3			0.88 (0.05)	0.12 (0.06)	0.23 (0.08)	0.15 (0.08)	−0.21 (0.11)	−0.34 (0.12)	−0.64 (0.06)	−0.93 (0.03)
LEAF				0.06 (0.04)	0.10 (0.05)	0.08 (0.08)	−0.17 (0.09)	−0.33 (0.11)	0.47 (0.10)	−0.71 (0.09)
Fall										
SEN1					0.99 (0.01)	0.59 (0.72)	0.94 (0.04)	−0.05 (ns)	0.02 (ns)	0.33 (0.06)
SEN3						0.88 (0.04)	0.90 (0.05)	−0.80 (0.13)	0.02 (ns)	0.35 (0.07)
LCOR5							0.94 (0.03)	−0.80 (0.09)	0.05 (ns)	0.55 (0.06)
LABS								0.86 (0.05)	0.12 (0.21)	0.78 (0.04)
Duration										
SENDU									−0.10 (0.16)	0.79 (0.13)
LEAFDU										0.47 (0.09)

*Note*: BUD1, bud phenology of score one; BUD3, bud break; LEAF, leaf out; SEN1, senescence of score one; SEN3, senescence of score three; LCOR5, leaf coloration; LABS, leaf abscission; SENDU, senescence duration, the period between leaf coloration and abscission; LEAFDU, leaf out duration, the period between bud break and LEAF; GL, growing season length, the period between LABS and BUD3; HT, tree height; the values were under a natural logarithm transformation; “ns” was not significant when standard error was more than the twice of genetic correlation absolute estimate value. Growing season length was positively correlated with the spring bud phenology (BUD1, BUD3) and the senescence during (SENDU), as well as the leaf abscission (LABS).

Multiple phenology stages were positively correlated with each other within the same season in spring and fall (*r*_*G*_ > 0.6). Leaf phenology duration was negatively related to bud break (score 3) as *r*_*G*_ = −0.64 ± 0.06 and positively related to growing season length (*r*_*G*_ = 0.47 ± 0.09). Senescence duration was not correlated with leaf out duration but was related to growing season length (*r*_*G*_ = 0.79 ± 0.13). The beginning of the phenology stage in each season (i.e., SEN3, BBK3) was negatively associated with the seasonal duration (SENDU, LAEFDU), but the ends of the phenology stage (i.e., LAB, LEAF) were positively related to the duration of the phenology. Avoiding earlier spring frost with a later bud break resulted in a shortened growing season length ( *r* _*G*_ = −0.9~−0.7); a later senescence led to a longer growing season ( *r* _*G*_ = 0.3–0.8).

### 3.2 Genetic parameters adaptive traits with quadratic trends over time

Among the 43 half‐sib families, for the spring bud break score 1 to 5 analyzed, the *V*_*A*_ decreased from 14,882 degree days^2^ to 7777 degree days^2^ during the period between May 19th and May 29th, with a quadratic trend over time (*V*_*A*_ *~b1 DoY* ^*2*^ *+ b2 DoY + c + e*, *R* ^2^ = 0.47). The *V*_*A*_ of leaf senescence ranged from 0 to 5 day^2^ after Sept. 8th, which followed a quadratic trend over time from phenology phase 1 to 7 within the 18‐day window (*R* ^2^ = 0.53). The *V*_*P*_ increased from 0 to 20 day^2^ with a quadratic relationship with the average DoY of the phenology (*R* ^2^ = 0.83). Narrow‐sense heritabilities (${\hat{h}}^{2}$) of spring phenology were ~0.9–1, while ${\hat{h}}^{2}$ of fall phenology was 0–0.39.

Lager genetic and phenotypic variances occurred at the beginning of spring phenology (*CV*_*A*_ and *CV*_*P*_ are 0.12~0.15) and after the beginning fall phenology (*CV*_*A*_ and *CV*_*P*_~0.01), or the early stage of hardening in September (*CV*_*A*_~0.10, *CV*_*P*_~0.25). The *CV*_*A*_ adjusted the overestimation of genetic variance partially due to the site environment. The *CV*_*A*_ of bud break calculated by heat‐sum was greater (~13%) than the fall leaf senescence (~1%) and bud break (~4%) measured by DoY. The population average of fall phenology correlated with latitude (*R* ^2^ = 0.7, *p* < .001) and MAT of provenance groups (*R* ^2^ = 0.3, *p* < .001).

Height ${\hat{h}}^{2}$ peaked at 0.46 (±0.14) at AB Foothills (Trial #33) and decreased to 0.12 (±0.08) at the BC trial. Genotype by environment effect of height was expected. A positive linear relationship was shown between ${\hat{h}}^{2}$ and extreme minimum temperature over 30 years (°C) of the trials (EMT) with a Pearson's correlation coefficient as 0.86 (*p* = .06); the relationship between ${\hat{h}}^{2}$ and trial latitude (N°) was not significant (r=−0.75, *p* = .1409). *CV*_*A*_ of tree height stays around 0.13. *CV*_*A*_ and *CV*_*P*_ of senescence followed quadratic functions over DoY (*R* ^2^ = 0.70, 0.92 respectively). The ${\hat{Q}}_{\mathrm{st}}$ of spring bud break was moderate to low (~0.2) indicating a low among population variation, while most of the senescence variation was identified among populations (${\hat{Q}}_{\mathrm{st}}$ ~ 0.7–0.9, Table 4). Also, spatial divergence occurs in fall phenology among populations based on a cline of the latitudinal gradient (*R* ^2^ = 0.7, *p* < .001) as the northern population shed leaves more than 10 days earlier than the southern (MN) populations.

### 3.3 Cell lysis and lethal temperature (LT50)

**FIGURE 2:**
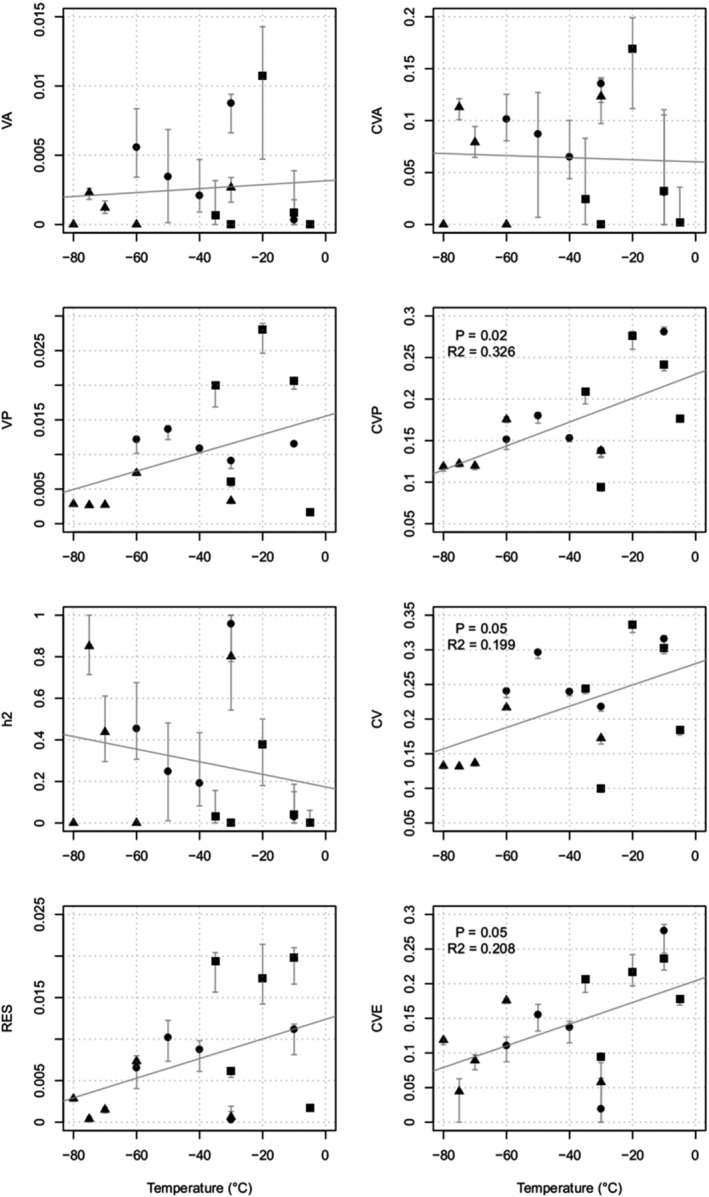
Correlation between the genetic parameters of cell lysis and the freezing temperature. The points were the average of estimates, while the bars were the upper (97.5%) and lower (2.5%) confidence intervals; the gray lines were the linear prediction of the parameters against temperatures. The right end of higher (e.g., −20 °C) was on Aug 28th (DoY 240, “■”); the middle of the temperature axis marked September 17th (DoY 260, “●”), and the coldest temperature was tested during Oct 17th (DoY 290, “▲”). “VA” was the additive genetic variance; “CVA” was the coefficient of additive genetic variance; “VP” was the phenotypic variance; “CVP” was the coefficient of phenotypic variance; “h2” was the narrow‐sense heritability; “CV” was the coefficient of variation of cell lysis; “RES” was the residual variance; “CVE” was the coefficient of variation of residual variance. Only the CVP, CV, and CVE demonstrated significant linear relationships (*p* < .05), while other genetic parameters were not significant at all of which no *p*‐Value was shown in the figure.

During the acclimation, the timing of reaching LT50 diverged among BC (North), AB (Middle), MN (South) provenance groups from −20 to −60°C. The *CV*_*A*_ of cell lysis ranged from 0 to 0.10, while *CV*_*P*_ ranged from 0.10 to 0.25 (Figure 2). On average, *CV*_*A*_ of the spring phenology and CL at the earlier stage were 4–6 times of fall senescence and the height (~12% in Table S4). The *CV*_*A*_ and *CV*_*P*_ of the early stage of frost hardiness in August were about 1–4 folds of the late stage in October and were 10 folds of that of the fall leaf senescence. The ${\hat{h}}^{2}$ demonstrated no linear trend versus freezing temperatures and varied around an average of 0.30 ranging from 0 to 0.9 among testing dates and temperatures (Figure 2). However, the phenotypic related parameters *CV*, *CVp*, and *CV*_*E*_ declined during the acclimation process (linear, *R* ^2^ = 0.2–0.3) under freezing temperatures from 0 to −80°C (Figure 2). For example, the *CVp* decreased (*R* ^2^ = 0.33, *p* = .02) over the time series from August 28 to Oct 17 (Figure 2). Higher genetic and phenotypic variances were expressed in the early and the intermediate process of hardening (above −40 °C) and such trend aligned with the temporal trend of LT50 and LT25 when the family average LT curves diverged (Figure S3). The *CVp* peaked at −10 to −20°C and the ${\hat{h}}^{2}$ spiked at −30°C and − 70°C. The selection temperatures for hardiness in fall can be from −10 to −40°C.

### 3.4 Suboptimality indicated no local optimal growth trend

The home‐site disadvantage of relative productivity demonstrated that provenance groups and families outperform the local when migrated to cooler species range and sites (Figure 4), that is, toward the lower EMT direction with a slope = −0.075 (*R* ^2^ = 0.52, *p* < .0001), and to the north with the slope = 0.026 (*R* ^2^ = 0.25, *p* < .0001). For the EMT transfer gradients, the correlation was a consistent trend for higher relative height, survival, and productivity when moving to cooler environments. Below the typical altitude of ~1000 m for aspen plantation, assisted migration potentially yielded ~40% more relative productivity compared to the local families when the EMT of sites were 4°C colder, despite the general depression of average growth under cooler climates at sites. No significant local adaptation trend was detected with the multiple environmental distances including the PC multivariate indices. The slopes of each transfer climatic distance ranged from −0.2 to 0.01 when distances were negative, which means a downward transfer, for the temperature‐related distances (e.g., MAT, MCMT, EMT, DD5) the movement was toward a cooler environment, except the altitude and latitude, where negative distances meant downslope or southward transfer (Figure 4). BC population and high‐level deme (#766) which was treated as the peripheral population for the plantation prescriptions showed low relative productivity and they drastically diverged from the prairie populations.

## 4 DISCUSSION

### 4.1 Suboptimality and the causes of adaptive traits

According to the growth‐climate optimality study by Rehfeldt et al. (2018), a difference between the ecological optimum and physiological optimum was expected to occur among the current western aspen populations, represented as a suboptimality when the transplants outperform the local (home‐site model, Figure 4 and Table S6). And such a non‐local adaptation trend was associated with the historical adaptation to a cooler climate that was evidenced in the genetic distinctiveness between spring and fall cold adaptation. The cooler environment that the study population tends to occur was offsetting from their physiological optimal during seed transfer (Rehfeldt et al., 2018); and in order to achieve the current optimal, fall frost has been a potential natural selection pressure of those populations compared to the spring frost while early frost damage trait showed high genetic variability for adaptation.

A “conservative” growth strategy reported led to a trade‐off due to an early phase and lower genetic control of fall senescence by compromising rapid height growth especially at warming sites that were historically released to allow species distribution post‐glaciation. Spring leaf phenology, as well as CL, harbored high adaptation potential and genetic control instead, which enables the study population to track the environmental cues including daylength, spring chilling degree days as well as the heat degree days. Despite raising frost risks, the moderate to high additive genetic controls and *CV*_*A*_ of CL suggested a greater potential for selection pressure in cooler environments with adequate capacities in the spring and fall hardiness traits to adapt to novel climatic conditions for *P. tremuloides*. A warming trend may have a limited advance of leaf phenology in spring for trees (Fu et al., 2015); and higher *CV*_*A*_ s provide the genetic variations for natural selection.

**FIGURE 3:**
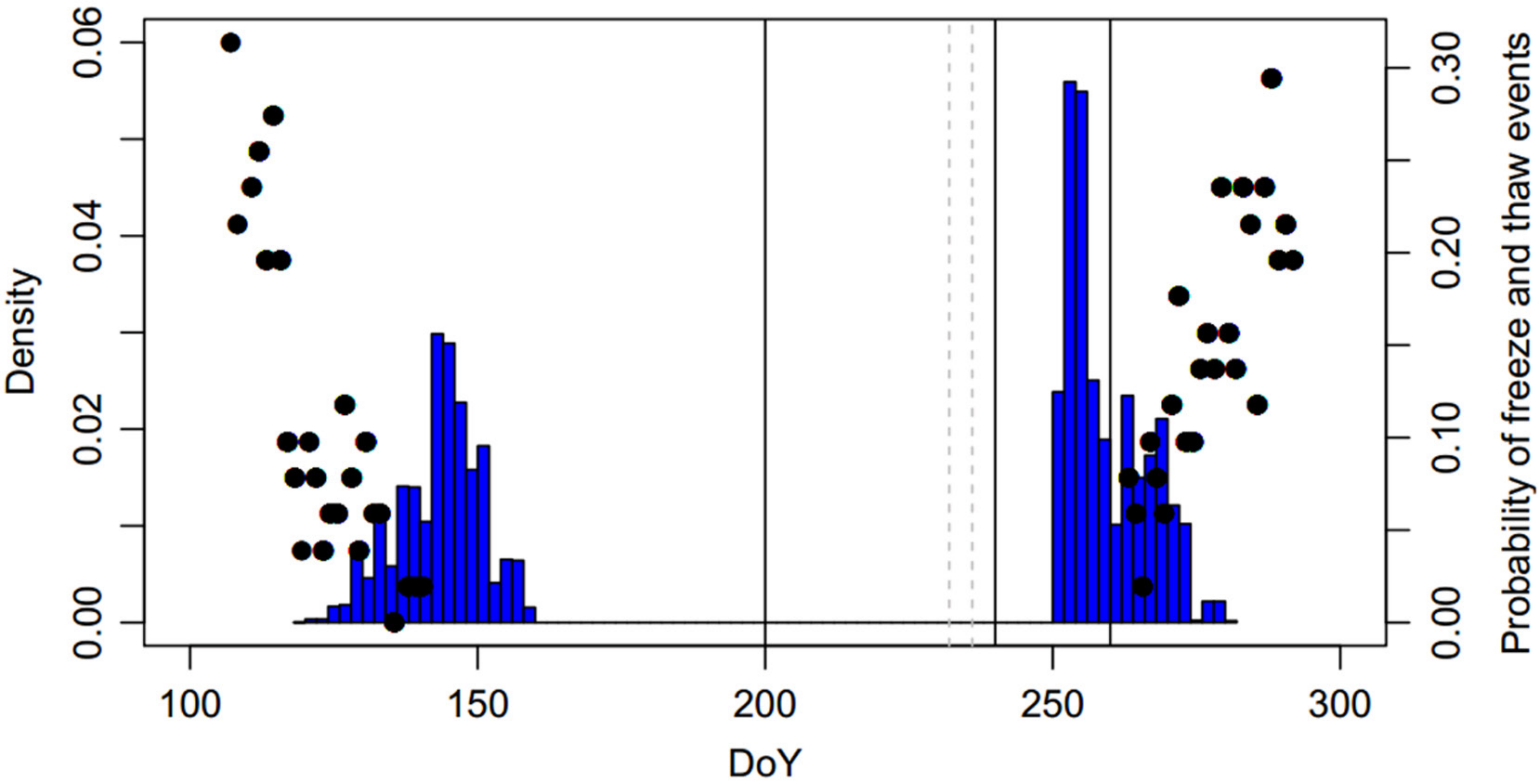
Histogram of adaptive traits in spring and fall (bars) versus the probability of freeze and thaw events (dots) from 1960 to 2010. The solid vertical lines showed the dates of hardiness measurement after August 2010 (DoYs 240 and 260). Senescence occurs about a week later than the hardening processes. The Climate Identifier of the weather station was 306,032, Athabasca 2 (54.821667° N, 113.539722° W, 626.3 m a.s.l.). Less than half of the adaptive trait occurs during the window of freeze–thaw events (before ~DoY 135 and after ~DoY 270). The spring and fall phenology were observed in two consecutive years and were not comparable in terms of the DoY and density.

Again, by avoiding the time window of frost and freeze–thaw major events, especially the fall frost (Figure 3), the trade‐offs between growth and senescence revealed a “conservative” grow strategies against the “non‐existing” harsh frost in fall and winter because such adaptation pattern was in line with the historical post‐glacial maximum climates (>14,000 years before present) with more frequent extreme low temperature rather than the current climatic conditions with elevated winter fall and winter temperatures. LT50 also demonstrated that the current population adapted to lower frost scenarios with early acclimation onset of last fall frost which was colder than the actual freezing temperature during the hardening onset in the field trial. The CL, physiological measurement of frost hardiness, expressed a broad genetic variability for selection (*CV*_*P*_ and *CV*_*A*_) especially during the onset period of hardening (~ −20°C for *CV*_*A*,_ 0 to −20°C for *CV*_*P*_). Thus, the ecological optimum tends to be warmer than the physiological optimum.

Within the current species range, the home‐site model based on the reciprocal transplant experiment suggested that the current population may improve relative growth potential when transplanted to a cooler species range (moving <−4°C of EMT distance) and this agrees with the previously reported direction of assisted migration (Gray et al., 2011). Degree‐days >5°C (DD5, *R* ^2^ = 0.19–0.31***) and latitudinal (*R* ^2^ = 0.25–0.27***) seed transfer to the cooler and northern sites are secondary options to improve the relative height and productivity. Those aligned with the climatic direction for movement for the EMT. Those factors above are not necessarily determinants of adaptability, though the non‐significant factors could not be excluded from the decision‐making process of planting site selection. We suggest the intraspecific seed transfer direction is promising with the relative productivity increase and successful mitigation stratifies such as assisted migration without maladaptation remain challenging for sustainable forestry and reforestation (Keenan, 2015). A local adaptation in the natural tree population reflects various biotic and abiotic environmental influences including genetic, geographic, climatic factors (Du et al., 2020; Hu et al., 2021; Keller et al., 2011; Wang et al., 2014; Zhang et al., 2019).

**FIGURE 4:**
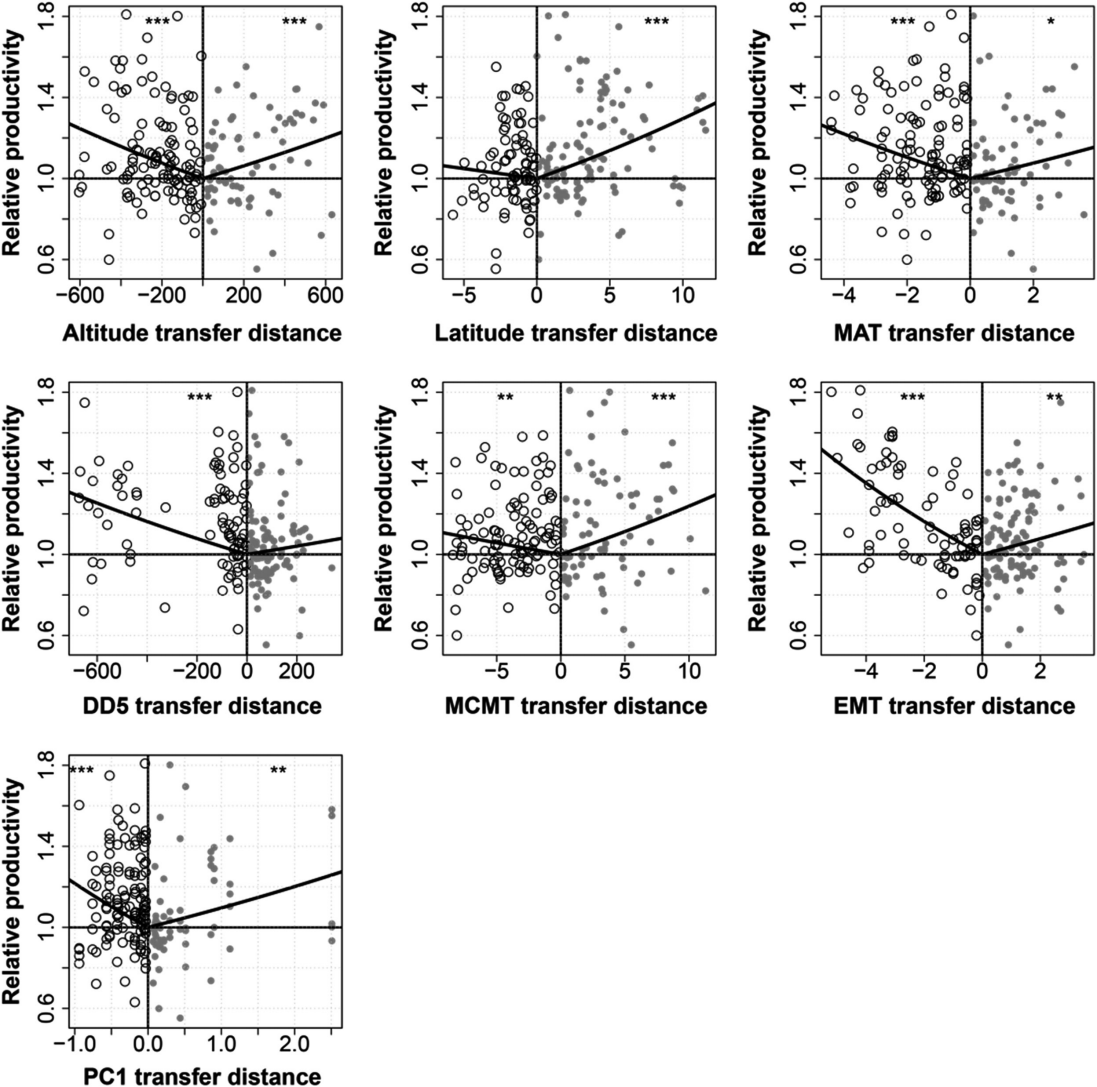
The home‐site models for relative productivity versus seven multiple environmental distances between the trial sites and the provenances (altitude, m; latitude, degree north; MAT, °C; DD5, degree days; MCMT, °C; EMT, °C; PC1 of 10 climatic variables). Due to the low relative productivity (<0.79) of BC provenances and provenance #766 from high latitude (1, 018 m) and the site range for plantation lower than 1000 m in general, which were treated as outliers excluded from analyses. Each provenance was listed in both upward (distance >0, the open icons) and downward (distance <0, the close icons) distances. The significant level of F test for each model was as *p* < .0001, “***”; <.001,“**”; <.01, “*”; <.05, “`”.

The trade‐off between cold tolerance for survival and growth cooperated with the disparity between ecological optimal versus the physiological optimal. Adequate genetic variation and capacity for adaptation align with the “cooling” direction for moderate assisted migration within the species range (ΔEMT<4°C), that is, spring leaf phenology and fall cell lysis. The disparity between ecological and physiological optima can fall around 4°C in terms of EMT, which can be translated to 5~10° northward in the study area (Figure 4). Relative growth potential was positively related to the transplant distance in the cooler direction of EMT depending on the site conditions though the absolute growth trend follows site conditions. BC population were adapted to the extreme condition in this study of which future transplant tests for improving the relative performance may fall in the neighboring north site. Besides the frost risks during seed transfer and the climatic distances, the local aspen growth potential is affected primarily by site quality and preparation, weed control and management, distance, and direction of latitudinal transfer. Excessive summer heat and drought during the growing season are limiting factors for aspen growth (Hogg et al., 2008; Morelli & Carr, 2011). Seed transfer from cooler to warmer sites needs caution due to maladaptation risks of the transplants in western Canada. In the southern contracting populations, that is, Texas, the United States, aspen stands are under multiple stresses such as drought and heat and wild fire disturbances (Nunneley et al., 2014) of which the sites are more difficult for natural regeneration than our study area; although the species was relatively heat tolerant compared to *Betula papyrifera* in the experimental settings (Teskey et al., 2015), sustaining and regeneration of the contracting populations are challenging under future climatic related stresses and disturbances.

Seed transfer from high latitude to lower latitudinal sites may not benefit the relative growth of transplants due to early bud set and growth cessation (Hall et al., 2007; Luquez et al., 2008; Soolanayakanahally et al., 2013; Zhang et al., 2019). The maladaptation risks varied according to the season: summer high temperature causes stress and risks of maladaptation of the local and transplants, while the fall frost leads to reduced risks as evidenced in frost hardiness and fall leaf phenology. Future field evaluation of the sensitivity to summer temperature is necessary.

Multiple biotic and abiotic factors can potentially limit the species range and tree mortality and productivity in forests (Anderegg et al., 2015; Keller et al., 2011; Ware et al., 2021; Worrall et al., 2013). Our study demonstrated low temperature as a main driver of the relative productivity change during seed transplant but it does not exclude other prevalent limiting factors such as drought (Michaelian et al., 2011), ozone and CO_2_ (Emily & Mark, 2013), population competition, pest, pathogen (Ruess et al., 2021), etc. Low temperature regulates the functions such as leaf phenology in spring (Körner et al., 2016) and in fall (Fracheboud et al., 2009) and the onset of the acclimation process (Wisniewski et al., 2014), which are strongly associated with the phenology of winter dormancy and eventually the species range. Trees that are distributed at the sites of temperature limits do not consistently adapt to a warmer climate, so long‐term monitoring will be necessary to confirm the survival and growth of the population at the frontier or peripheral sites.

### 4.2 Dissimilarity of adaptive trait genetic variations in spring and fall

Genetic variability of spring leaf phenology such as bud burst and leaf out was likely driven by the local environment of the provenance and plantation site (McDonough MacKenzie et al., 2018). Micro‐evolution over the long‐term shapes the current spring phenology of trees, which is tied with the trade‐off between late flushing and long growing season length (Körner et al., 2016). Spring frost is not a major concern here due to the safety of spring leaf out timing that avoids the freezing window (Figure 3), which agrees with the trend of deciduous trees in Europe (Körner et al., 2016). A warming climate could modify the spring phenology and leaf development of broadleaf trees depending on the inter‐annual weather conditions (Wisniewski et al., 2018). Leaf senescence phenology has a short‐day trigger for onset and warm night temperature cue for senescence duration before dormancy (Tanino et al., 2010). This is limited to a narrow window near autumn equinox for *P. tremuloides* (Schreiber, Ding, et al., 2013). In *P. tremula*, the northern populations are expected to have a greater selection response under climate than the contracting populations in the south with more chances of adaptational‐lag (Ingvarsson & Bernhardsson, 2020). No strong trade‐offs between spring frost avoidance and growth were identified compared to the fall phenology previously in other *Populus* species (Hall et al., 2007; Luquez et al., 2008). Spring leaf phenology expressed spiking *CV*_*A*_ and *h* ^*2*^ but did not contribute to the variation of growth or productivity compared to the fall senescence trait in *P. tremuloides*.

Boreal species such as *P. tremuloides* have deep chilling requirements before spring bud burst and are likely to be affected by the warming trend in early spring (Harrington & Gould, 2015; Man et al., 2017). Chilling conditions are usually linked to the long‐term coldness of the provenance regions. Within the species range, chilling requirements are usually met when the population moved to a cooler site; thus, the up‐slope and north‐ward seed transfers assist the plantation to meet chilling requirements of their provenance at the new sites, especially under a warming spring trend. If the chilling requirement is not met, the timing of bud burst tends to lag even under the triggering heat‐sum conditions (Man et al., 2017). Other environmental factors delay budbreak include dryness in winter and spring (Li et al., 2010). We suggest that the genetic variation of spring leaf phenology could be translated as the family and clonal variance when the chilling requirement is met at most of the sites within the species range (Man et al., 2017), and the heat‐sum trigger marks the expressed genetic variation of spring leaf phenology. Fall phenology may have less genetic potential especially under diversifying selection pressure (i.e., high pressure in northern BC population but lower in the southern populations), but they still avoid the frost window well before late October (Figure 3) (Schreiber, Ding, et al., 2013). Leaf senescence marks the end of leaf photosynthesis and relates to the relocation of nutrient from leaves to the trunk, which was triggered by photoperiod in boreal broadleaf trees (Fracheboud et al., 2009; Keskitalo et al., 2005; McKown et al., 2013). The fall phenology showed much lower *CV*, *CV*_*A*_, *CV*_*P*_ (Table 3), and skewed distribution (Figure 3) subjected to stronger selection and adaptation to the local fall environment.

**TABLE 3:** Trait means, genetic parameters of adaptive traits

Trait	Score	Mean	*V*_*f*_	*V*_*pop*_	*V*_*e*_	*V*_*A*_	*CV*_*A*_	*V*_*P*_	*CV*_*P*_
Bud break	1	733 (3)	1501 (398)	4529 (3103)	4385 (208)	6005 (1591)	11	5887 (449)	10
Degree‐days	1.5	767 (4)	3721 (945)	7763 (5447)	6725 (319)	14,882 (3781)	16	10,446 (998)	10
	2	751 (3)	2056 (536)	3983 (2832)	5757 (273)	8225 (2144)	12	7814 (602)	12
	2.5	809 (4)	3324 (843)	8004 (5534)	6105 (290)	13,297 (3372)	14	9429 (892)	12
	3	806 (3)	2635 (669)	4755 (3373)	5521 (262)	10,541 (2678)	13	8156 (719)	11
	3.5	845 (3)	3006 (764)	7188 (4976)	5496 (261)	12,025 (3056)	13	8502 (808)	11
	4	847 (3)	2375 (611)	4277 (3038)	5008 (238)	9502 (2443)	12	7383 (656)	10
	4.5	882 (3)	2807 (709)	6321 (4403)	5130 (243)	11,229 (2837)	12	7938 (750)	10
	5	896 (3)	1944 (496)	2491 (1844)	3900 (185)	7777 (1984)	10	5844 (529)	9
Senescence	1	251.7 (0.0)	0.0 (0.0)	0.2 (0.1)	0.2 (0.0)	0.0 (0.0)	<1	0.2 (0.0)	<1
DoY	1.5	252.8 (0.0)	0.0 (0.0)	0.8 (0.6)	1.2 (0.1)	0.1 (0.1)	<1	1.2 (0.1)	<1
	2	253.4 (0.0)	0.0 (0.0)	0.7 (0.5)	0.7 (0.0)	0.2 (0.1)	<1	0.7 (0.0)	<1
	2.5	254.5 (0.1)	0.0 (0.0)	2.0 (1.4)	2.0 (0.1)	0.1 (0.2)	<1	2.0 (0.1)	1
	3	256.0 (0.1)	0.2 (0.1)	6.4 (4.1)	3.5 (0.2)	0.7 (0.4)	<1	3.7 (0.2)	1
	3.5	257.2 (0.1)	0.5 (0.3)	11.3 (7.3)	7.3 (0.3)	1.9 (1.1)	1	7.7 (0.4)	1
	4	258.7 (0.1)	0.4 (0.2)	11.4 (7.3)	5.9 (0.3)	1.7 (0.8)	1	6.3 (0.4)	1
	4.5	260.4 (0.2)	1.0 (0.4)	21.4 (13.8)	10.2 (0.5)	3.8 (1.7)	1	11.2 (0.7)	1
	5	262.3 (0.1)	0.5 (0.3)	16.8 (10.8)	9.0 (0.4)	1.9 (1.2)	1	9.5 (0.5)	1
	5.5	263.1 (0.2)	1.3 (0.5)	25.7 (16.6)	12.1 (0.6)	5.2 (2.2)	1	13.4 (0.8)	1
	6	265.8 (0.1)	0.1 (0.2)	18.3 (11.7)	9.2 (0.4)	0.5 (0.8)	<1	9.4 (0.5)	1
	6.5	267.6 (0.2)	0.5 (0.4)	32.2 (20.6)	18.2 (0.9)	1.8 (1.7)	1	18.7 (1.0)	2
	7	270.0 (0.1)	0.2 (0.2)	23.0 (14.6)	10.7 (0.5)	0.7 (0.8)	<1	10.9 (0.5)	1

*Note*: 0.0 (0.0) means the estimates and standard error <0.01. The standard errors were in parenthesis; *CV*_*A*_ was the additive genetic coefficient of variation (%), and *CV*_*P*_ was the coefficient of phenotypic variation (%).

**TABLE 4:** The ${\hat{h}}^{2}$ and ${\hat{Q}}_{\mathrm{st}}$ of adaptive traits

Trait	Score	${\hat{h}}^{2}$ ^a^	S.E.	${\hat{Q}}_{\mathrm{st}}$	S.E.
Bud Break	1	1.02	0.20	0.27	0.15
Degree‐ days	1.5	1.42	0.24	0.21	0.12
	2	1.05	0.21	0.19	0.12
	2.5	1.41	0.24	0.23	0.13
	3	1.29	0.23	0.18	0.11
	3.5	1.41	0.24	0.23	0.13
	4	1.29	0.23	0.18	0.11
	4.5	1.41	0.24	0.22	0.13
	5	1.33	0.23	0.14	0.09
Senescence	1	0.29	0.14	0.65	0.19
DoY	1.5	0.06	0.10	0.85	0.25
	2	0.23	0.12	0.68	0.19
	2.5	0.07	0.10	0.88	0.17
	3	0.20	0.11	0.82	0.13
	3.5	0.24	0.13	0.75	0.16
	4	0.28	0.13	0.77	0.14
	4.5	0.34	0.14	0.74	0.15
	5	0.20	0.12	0.81	0.14
	5.5	0.39	0.15	0.71	0.16
	6	0.05	0.08	0.95	0.08
	6.5	0.10	0.09	0.90	0.10
	7	0.07	0.07	0.94	0.07

^a^ Estimated ${\hat{h}}^{2}$of bud break may exceed 1.00 by using the half‐sib family variance structure.

Our ${\hat{h}}^{2}$ estimate of bud break was at a higher range (>1), partially due to the coefficients of genetic family models to estimate the additive genetic variance (i.e., 4), full‐sib families occurrence in the progeny within the half‐sib families, and limited environment variance to precisely estimate the genetic and phenotypic variances. Howe et al. (2003) suggested that the heritability of bud flush was higher than bud set. Here, we demonstrated lower heritability of senescence and later stage hardiness at each testing DoY (i.e., cell lysis) that were averaged as ~0.4, compared to the spring leaf phenology (0.9–1). Potential full‐sib families may exist among the offspring of 43 mother trees which may not be randomly mated half‐sib families, and this may lead to an overestimation of the additive genetic variance (*V*_*A*_). The realized growing season was determined by the period from the terminal bud break to leaf senescence, which is a proximation of the growing season length. The earlier stage of spring leaf phenology (in mid‐May) also represented the additive genetic variance level of bud break, compared to the previous arbitrary bud break stage of a single score (Schreiber, Ding, et al., 2013).

The leaf flushing date and growing season diverged more among families than between populations, so we suggest a weak frost selection pressure in spring in the study area. The alternative mutualism between herbivore and spring leaf phenology demonstrated earlier and later bud break matched the timing of *Malacosoma disstria* development, a major boreal pest of aspen forests, due to the bud break phenology coinciding with the egg hatch (Parry et al., 1998). Thus, selection pressure might come from factors such as insect herbivores rather than simple climatic drivers such as spring frost. In other tree species, leaf phenology was found associated with the herbivore population dynamics (Aide, 1992; Gherlenda et al., 2016). Though the insects can synchronize the life history with the hosts' phenology (Ju et al., 2017), a wider phenology time window of spring bud break and leaf‐out (increasing *CV*_*A*_, *CV*_*P*_, and *V*_*A*_) will contribute to a potential buffer effect to the foliage loss due to the herbivore during the nutrient‐limited period of stands by mismatching the phenologies between host trees and insects.

### 4.3 Population diverged in cold adaptation and fall leaf phenology

Howe et al. (2003) suggested the differential natural selection among populations of *P. tremuloides* previously. Here, we dissected the extent of intra‐specific selection responses on spring and fall cold adaptation traits and found stronger population differentiation on fall phenology traits than on the spring traits. We demonstrated the species‐range estimation of ${\hat{Q}}_{\mathrm{st}}$ for the continuums of spring (0.2–0.3) and fall leaf phenology (0.6–0.9) and identified higher population differentiation of fall phenology than the spring phenology. Therefore, natural selection was prevailing to cause population differentiation of fall phenology rather than the spring phenology (Liu & El‐Kassaby, 2019).

When ${\hat{Q}}_{\mathrm{st}}$ of quantitative traits exceeds the magnitude of *F*_*st*_, the traits are likely under diversifying selection for phenotypic traits (Savolainen et al., 2007). In *Populus tremuloides*, subdivided genetic population structure was not strong (Latutrie et al., 2016). Bud flush was weaker in adaptive divergence among populations than other adaptive traits such as drought tolerance. In other species, the environmental drivers of adaptive divergence include heat, drought, and moisture‐related indices (Csilléry et al., 2020). For bud flush, previously reported ${\hat{Q}}_{\mathrm{st}}$ (~0.14) was lower than our results partially due to the limited sampling range of the species (Thomas et al., 1997).

In *P. tremuloides*, the neutral marker studies demonstrated an *Fst* as ~0.03 which means 97% of the genetic variation was within populations partially due to the wind‐pollination and outcrossing reproduction mode that reduced the population differentiation at the molecular marker level. We found the trend of divergent selection was likely to cause the population divergence in fall phenology trait. Similarly as reported in *P. tremula*, a discernable adaptive divergence of phenology was uncovered across the latitudinal gradient with ${\hat{Q}}_{\mathrm{st}}$ > 0.5 while *F*_*st*_ ~ 0.015 (Hall et al., 2007). Such a trend was not uncommon and was probably due to the non‐additive gene actions (Goudet & Büchi, 2006; Hall et al., 2007); and previous regional studies of genetic parameters demonstrated moderate to low dominance and epistasis genetic effects in growth and phenology traits in an Alberta population (Ding et al., 2020).

There are several factors compromising the precision of estimating population divergences such as the lack of an unbiased evaluation of ${\hat{Q}}_{\mathrm{st}}$, unknown dominance and epistasis genetic effects (Hall et al., 2007), and the lack of neutral loci differentiation (*F*_*st*_) for the total populations including all extreme demes (Whitlock, 2008). Because the within‐population variance was estimated based on half‐sib family variance, the ${\hat{Q}}_{\mathrm{st}}$ could be slightly underestimated while the ${\hat{h}}^{2}$ is overestimated. For the current study area, our estimate was adequately represented. Other factors may have biased ${\hat{Q}}_{\mathrm{st}}$ estimates such as inbreeding, drift, and maternal effects. (Yang et al., 1996).

### 4.4 Adaptive traits correlation and trade‐off between growth and adaptation

Multiple studies in deciduous and conifer species showed the trade‐off between cold adaptation to frost and growth in the boreal species (McKown et al., 2014; Sebastian‐Azcona et al., 2019). We confirmed the fall phenology of lower variability was positively correlated to height growth ( *r* _*G*_ ~ 0.7–0.8) while spring leaf phenology had less correlation with growth ( *r* _*G*_ ~ −0.2) based on the species‐range wide samples. We also confirmed that across the species range in western Canada, the increasing growth was associated with late leaf senescence and no survival depression was found when families showed a late onset of leaf senescence; and this trend was previously reported in a within‐population study (Ding et al., 2020). The bud break of score one and leaf coloration showed a coincidence of late bud break with lagging coloration that was the only correlated spring and fall phenological stages. It was likely a protection strategy to avoid either spring or fall frost by maintaining the growing season, but other phenology stages were not correlated among the two seasons. Height growth was genetically correlated with the growing season length (*r*_*G*_ = 0.5).

### 4.5 Acclimation demonstrated with LT and genetic adaptation to frost

The lethal damage temperatures (LT) linked to the acclimation to winter dormancy (Figure S3) and *CV*_*P*_ of CL were positively correlated with the declining temperature (*R* ^2^ = 0.33) to track the acclimation weather condition (Figure 2). The LT50 dropped during the hardening and also expressed the process of dehydration in tissue as reported previously (Lennartsson & Ogren, 2002). The associated genetic and phenotypic variances declined when freezing temperature dropped (Figure 2). The freezing temperature was a potential predictor of the *CV*_*P*_ and *CV*_*E*_ and explained 33% of the *CV*_*P*_ variances but not in the case of ${\hat{h}}^{2}$ or *CV*_*A*_ (*R* ^2^ < 0.1). The genetic parameters of leaf phenology and CL demonstrated different trends as quadratic and linear, respectively, during the process of breaking growth initiation and acclimation. Here, the CL was measured among 48 genotypes from six families compared to over 6000 trees for the height, so CL was not used to infer the genetic correlation with growth.

During the later phase of freezing (<−70°C), the twig could accumulate sugar to raise the tolerance to freezing damage and *CV*_*P*_ dropped substantially. LT was negatively correlated with the soluble carbon hydrate concentrate in the tissues of temperate trees (Morin et al., 2007). The additive genetic variance (*V*_*G*_) was not correlated with the freezing temperature during acclimation that was only expressed as a moderate to a high level at certain stages of the freezing test. A leap value of *CV*_*A*_ at −75°C caused the high ${\hat{h}}^{2}$ and the reasons can lie in the higher standard error of the ${\hat{h}}^{2}$, sampling error associated with the date or temperature treatment, tissue quality, and experiment conditions. To further confirm −75°C as a freezing temperature for CL trait selection, more repeated tests with extended family sampling are necessary.

Previous studies demonstrated the impact of repeated freeze–thaw events in the spring and fall which caused vessel embolism, and therefore, worsened the frost injury and eventually advanced the dieback and mortality rate over winter (Schreiber, Hamann, et al., 2013). Although the freeze–thaw events in spring and fall both occur outside the median and density peak of phenology (Figure 3), there was ~10‐day overlap between the phenology and freeze–thaw time window with the event probability >10% both in spring and fall. More genetic gain could be achieved in the early spring bud break and more height gain can be realized when families of late senescence are utilized. Though CL provided greater genetic gains for breeding and selection, cautious frost damage assessment will be necessary over the long term when the freezing‐thaw impact can exceed the trees' frost tolerance.

We found that *Populus tremuloides* demonstrated a different (warmer) ecological optimum than the physiological optimum, which agrees with Rehfeldt et al. (2018)’s finding in *Pinus contorta*. *Populus tremuloides* is a cold tolerate boreal deciduous tree distributed over a wide area (Girardin et al., 2022). Conservative growing strategies of avoiding frost damage were demonstrated with earlier senescence and onset of hardiness than the real frost temperature occurrence. This demonstrated a trade‐off between early senescence versus growth. Assisted migration to cooler sites (−4°C EMT) is promising, with an increase in 40% of relative productivity within this study area. The current seed transfer experiment direction parallels that of population recolonization after the last glacial maximum so that the potential adaptational‐lag will be decreased when the southern families such as the Minnesota provenance group are deployed in the northern sites of the study area.

The correlated response of early bud break and late senescence due to height selection was first confirmed in the western Canadian population collections. Growth variation was positively correlated with lagging fall leaf coloration and abscission (genetic correlation *r*_*G*_ = 0.80). Spring bud break showed a weaker population divergence than fall leaf senescence. The coefficient of additive genetic variance (*CV*_*A*_) in spring phenology and frost hardiness (cell lysis) were about 4‐ and 6‐fold of leaf senescence. The coefficient of phenotypic variation (*CVp*) for cell lysis ranged from 0.10 to 0.28, and shrank during acclimation. Freezing temperature (°C) was a predictor of the *CV*_*P*_ for cell lysis (*R* ^2^ = 0.33). The *V*_*A*,_ *CV*_*A*_, and *CV*_*P*_ of the spring leaf phenology demonstrated quadratic trends (*R* ^2^ = 0.44–0.74) over time, while *CV*_*P*_ and *CV*_*A*_ for cell lysis followed a linear trend during the acclimation.

Assisted migration is possible by using hardy provenances and families especially when the plantation site is cooler than the provenance site. Under a warming climate, the current populations are experiencing more benign winter temperatures than were experienced historically during species range expansion. For tree improvement, long‐term field screening of fast growers with known frost tolerance in spring and fall is still necessary due to the selection response of spring phenology and early‐stage cell lysis in fall. Growth cessation, fall leaf senescence, spring phenology, and early frost hardiness are all potential traits to be targeted for breeding for future reforestation.

## AUTHOR CONTRIBUTIONS

**Chen Ding:** Conceptualization (equal); data curation (equal); formal analysis (equal); investigation (equal); methodology (equal); software (equal); supervision (equal); validation (equal); visualization (equal); writing – original draft (equal); writing – review and editing (equal). **Jean S. Brouard:** Conceptualization (equal); data curation (equal); funding acquisition (equal); investigation (equal); resources (equal); validation (equal); writing – original draft (equal); writing – review and editing (equal).

## FUNDING INFORMATION

The research was supported by the grant from Andreas Hamann's research group and the in‐kind support from Isabella Point Forestry Ltd. and Western Gulf Forest Tree Improvement Program. Funding was provided by an NSERC/Industry Collaborative Development Grant CRDPJ. 349,100‐06 and an NSERC Discovery Grant RGPIN‐330527‐13 through the Government of Canada. We thank Alberta‐Pacific Forest Industries, West Fraser Grande Prairie & High Level (previously Ainsworth Engineered Canada LP), Mercer Peace River Pulp (formerly Daishowa‐Marubeni International Ltd.), Western Boreal Aspen Corporation, and Weyerhaeuser Company Ltd. for their financial and in‐kind support.

## CONFLICT OF INTEREST

The author declares that they have no conflict of interest.

## Supporting information


Data S1

## Data Availability

Data of adaptive traits, weather condition, home‐site model, and LT50 are available in a publicly accessible repository. https://doi.org/10.5061/dryad.g79cnp5sw.
