# Supplementary material for: Assisted migration is plausible for a boreal tree species under climate change: A quantitative and population genetics study of trembling aspen (Populus tremuloides Michx.) in western Canada
Source: Ecol Evol. 2022 Oct 5;12(10):e9384. doi: 10.1002/ece3.9384 (PMC9534759; doi:10.1002/ece3.9384)
Supplement: Supplementary file 1 — Data S1 [file ECE3-12-e9384-s001.docx]

**Supporting Information**

Additional supporting information may be found online in the Supporting Information section.

Table S 1. Experimental design of five common garden trials including reps, plots, families (treatment), total trees tested, survival, as well as tree height and diameter at breast height (DBH) measurement of age 9.

| Test site | Replicate | Families | Trees | Survival | Height (m) | DBH (cm) |
| --- | --- | --- | --- | --- | --- | --- |
| British Columbia #70 | 6 | 43 | 1290 | 0.87 | 3.0 | 2.3 |
| AB North #10 | 6 | 43 | 1350* | 0.94 | 5.1 | - |
| AB Central #60 | 6 | 43 | 1290 | 0.92 | 5.4 | 6.7 |
| AB Foothills #33 | 6 | 43 | 12751 | 0.77 | 3.3 | 2.5 |
| Saskatchewan #90 | 6 | 43 | 1290 | 0.63 | 3.4 | 2.8 |

Note: *) 3 families were tested twice in on replicate; 1) one family was missing in one replicate.

Weather station (306032) was chosen for AB Central #60

*
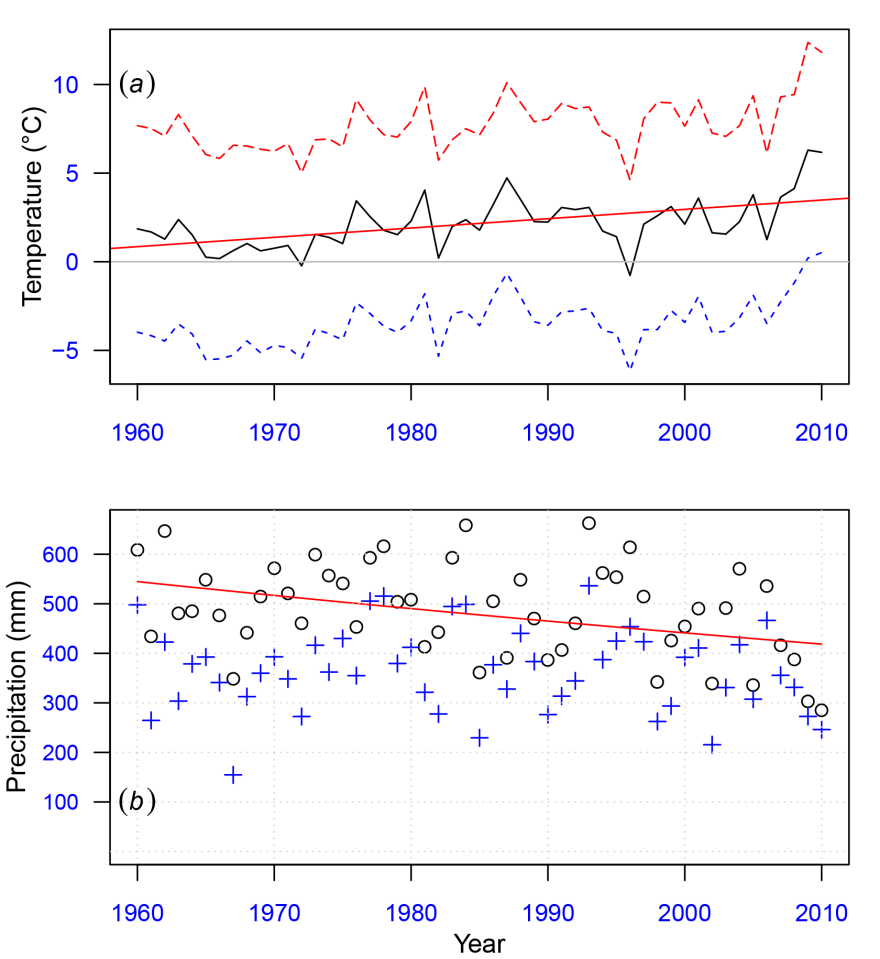
*

Fig. S 2. Relationship between climatic variables (air temperature, °C, precipitation, mm) and years from 1960 to 2010. (a) The upper line was the annual maximum temperature; the middle curve was the annual mean temperature. And the straight regression line goes through the curve of mean annual temperature. The regression slope of temperature change by year was +0.053 °C/Year, and the correlation was significant (Adjusted *R2*=0.28, *p-Value*<0.0001). The grey lines indicated 0 °C. The lower dashed curve was the annual minimum temperature. (b) Each circle represented the annual total precipitation at the trial, and the cross indicates the precipitation as rain per year. The precipitation was decreasing by year at an approximate rate of -2.60 mm/Year (log-transformed precipitation), and the correlation was significant (Adjusted *R2*=0.13, *p-Value* = 0.0057).


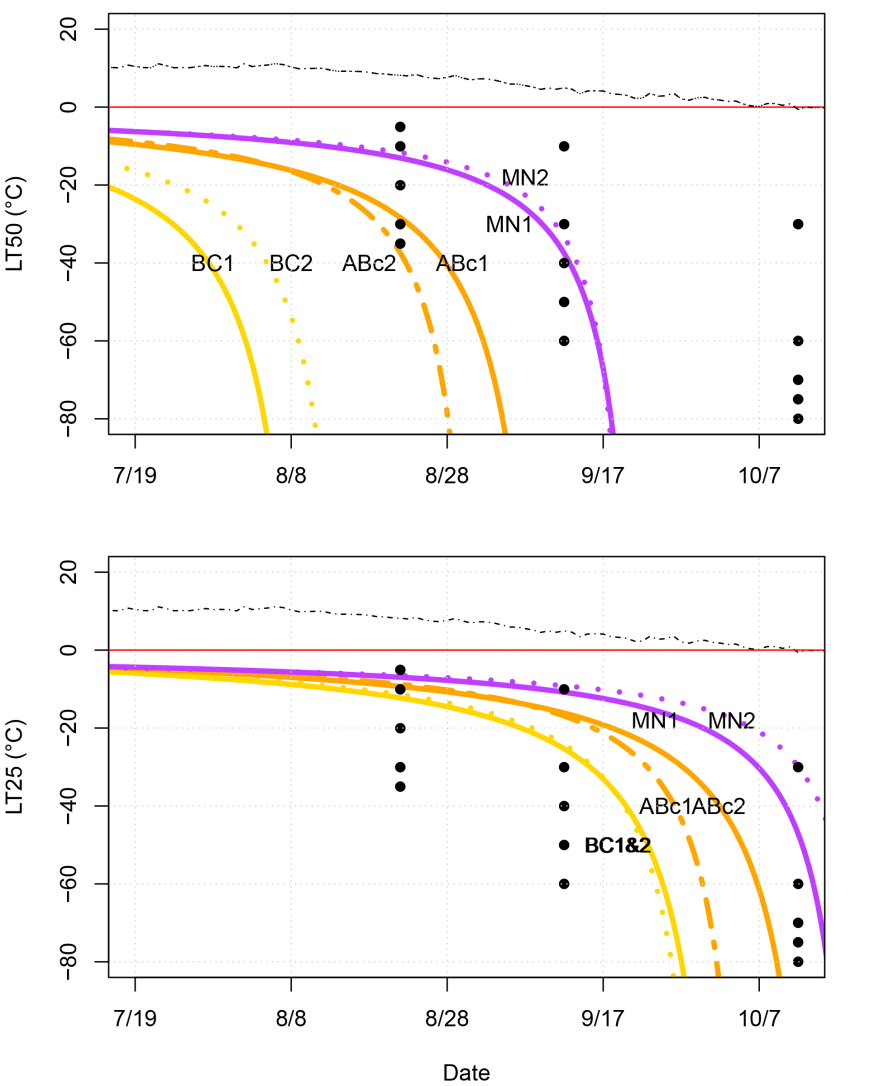


Fig. S 3. Temporal change in lethal temperature causing 50% (LT50) and 25% injury (LT25) for aspen.

Note: Estimates of cold hardiness were truncated at the minimum test temperature (°C). The dashed line above was the average minimum daily temperature at the nearest meteorology station (1960-2010). The vertical points were artificial freezing test temperatures on sampling dates. Each LT curve represents a provenance. The provenances from the left (less vulnerable) to the right (more vulnerable) were BC 1, BC2, Alberta Central (ABc) 1 and 2, Minnesota (MN) 1 and 2. July 19th  was DoY 200; Aug 28th was DoY 240; Sept 17 was DoY 260; Oct 17th was DoY 290. The solution and model parameters were shown in Table S 5.

Table S 4. Averages of trait measurement at trials, variance components, and narrow-sense heritability estimates with SE (in parentheses) of age 9.

|  |  | Variance components | | | Heritability | *CVA* (%) |
| --- | --- | --- | --- | --- | --- | --- |
| Trait (test site) | Average  ( m) | S (SE) | B × S (SE) | Error (SE) | $\hat{h}^{2}$ (SE) |
| Height (BC #70) | 3.0 | 0.03 (0.02) | 0.16 (0.04) | 0.87 (0.04) | 0.12 (0.08) | 12 |
| Height (nAB #10) | 5.1 | 0.10 (0.03) | 0.09 (0.03) | 0.87 (0.04) | 0.37 (0.12) | 12 |
| Height (cAB #60) | 5.4 | 0.13 (0.04) | 0.14 (0.04) | 0.96 (0.04) | 0.42 (0.13) | 13 |
| Height (ABf #33) | 3.3 | 0.09 (0.03) | 0.05 (0.02) | 0.65 (0.03) | 0.46 (0.14) | 18 |
| Height (SK #90) | 3.4 | 0.05 (0.03) | 0.17 (0.04) | 0.70 (0.04) | 0.20 (0.12) | 13 |

Note: S, family (within provenance group) effect; BxS (within provenance group), Block x family (within provenance group); Error the within plot random residual; VA = 4VS ; VP = VS + VBxS + VError; $\hat{h}^{2}$=VA/VP; Averages of height at trials were in meters, variance components, and narrow-sense heritability estimates ($\hat{h}^{2}$) with SE (in parentheses). The SE of variance components and $\hat{h}^{2}$were calculated with the delta method. The model was solved with PROC MIXED in SAS. The additive genetic effect could be overestimated because there were potential full-sib families within the open-pollinated families. The study does not have additional pedigree data to quantify the overestimation and the half-sib family structure was well defined.

Table S 5. Solutions of the fitted model genetic variation of lethal temperature (LT).

| Effects | Degrees of freedom | Denominator degrees of freedom | *F-Value* | *P* |
| --- | --- | --- | --- | --- |
| Provenances | 6 | 661 | 149.48 | <.0001*** |
| (1/t)*(1/t)* Provenances | 6 | 661 | 0.56 | 0.7636 |
| Sampling date* Provenances | 6 | 661 | 114.95 | <.0001*** |
| (1/t)* Provenances | 6 | 661 | 10.7 | <.0001*** |
|  |  |  |  |  |
| Provenances | *b0* | *b1* | *b2* | *b3* |
|  | (Provenances) | (1/t)* Provenances | (1/t)*(1/t)* Provenances | Date* Provenances |
| 759 (BC1) | 3.0938 | 5.6158 | 7.7478 | -0.00935 |
|  | *** | . |  | *** |
| 760 (BC2) | 3.5019 | 5.4192 | 3.5854 | -0.01092 |
|  | *** | * |  | *** |
| 775 (cAB1) | 4.5658 | 6.4203 | 4.7133 | -0.01452 |
|  | *** | ** |  | *** |
| 776 (cAB2) | 4.6248 | 8.8394 | 13.1837 | -0.01423 |
|  | *** | *** |  | *** |
| 789 (MN1) | 5.8107 | 7.7399 | 1.2205 | -0.01806 |
|  | *** | *** |  | *** |
| 791 (MN2) | 5.2414 | 4.8014 | -10.8405 | -0.01603 |
|  | *** | . |  | *** |
|  |  |  |  |  |
| Fit statistics | |  |  |  |
| -2 Res Log Likelihood | 9.1 |  |  |  |
| AIC | 11.1 |  |  |  |
| AICC | 11.1 |  |  |  |
| BIC | 15.6 |  |  |  |

Note: t was temperature; Provenances was the fixed effect of 6 provenances; data was the sampling date effect; b0 to b3 were the fixed provenance coefficients; the model was fitted as a quadratic function.

Table S 6. Regression results of home-site models for relative height, productivity, and survival across multiple environmental distances between the trial sites and the provenances.

| Relative growth | Distance<0 | |  |  |  | Distance>0 | |  |
| --- | --- | --- | --- | --- | --- | --- | --- | --- |
|
|  | Distance | Coefficients | *Adj. R2* |  |  | Coefficients | *Adj. R2* |  |
| Relative | *Altitude* | -0.0002 | 0.1109 | *** |  | 0.0002 | 0.1049 | ** |
| height | **DD5** | **-0.0003** | **0.3080** | *** |  | -0.0001 | -0.0064 |  |
|  | **EMT** | **-0.0444** | **0.4619** | *** |  | 0.0269 | 0.0816 | ** |
|  | *Latitude* | 0.0090 | 0.0206 | . |  | **0.0182** | **0.2677** | *** |
|  | MAT | -0.0332 | 0.1699 | *** |  | 0.0085 | -0.0036 |  |
|  | MCMT | -0.0049 | 0.0168 | . |  | 0.0096 | 0.0651 | * |
|  | PC1 | -0.1121 | 0.0956 | *** |  | 0.0671 | 0.1462 | *** |
|  |  |  |  |  |  |  |  |  |
| Relative | *Altitude* | -0.0004 | 0.1925 | *** |  | 0.0003 | 0.1415 | *** |
| productivity | DD5 | -0.0004 | 0.1940 | *** |  | 0.0002 | 0.0038 |  |
|  | **EMT** | **-0.0754** | **0.5173** | *** |  | 0.0374 | 0.0686 | ** |
|  | *Latitude* | -0.0095 | -0.0010 |  |  | **0.0259** | **0.2511** | *** |
|  | MAT | -0.0497 | 0.1688 | *** |  | 0.0366 | 0.0544 | * |
|  | MCMT | -0.0111 | 0.0539 | ** |  | 0.0214 | 0.1270 | *** |
|  | PC1 | -0.1963 | 0.1219 | *** |  | 0.0918 | 0.1118 | ** |
|  |  |  |  |  |  |  |  |  |
| Relative | *Altitude* | -0.0002 | 0.1594 | *** |  | 0.0001 | 0.0402 | * |
| survival | DD5 | -0.0001 | 0.0082 |  |  | 0.0003 | 0.0390 | * |
|  | **EMT** | **-0.0310** | **0.3420** | *** |  | 0.0104 | 0.0012 |  |
|  | *Latitude* | -0.0185 | 0.0494 | * |  | 0.0077 | 0.0817 | ** |
|  | MAT | -0.0165 | 0.0519 | ** |  | 0.0281 | 0.0638 | * |
|  | MCMT | -0.0062 | 0.0409 | * |  | 0.0117 | 0.0969 | ** |
|  | PC1 | -0.0842 | 0.0579 | ** |  | 0.0247 | 0.0054 |  |

Notes: BC provenances and provenance 766 (high elevation) were not included due to the high mortality and inferior growth at the transferred sites. The absolute coefficients and their standard errors (SE) of distances of both upward (distance>0) and downward (distance<0) were listed including the R square adjusted. The distances of altitude and latitude were not in the same direction as other variables from mild climates to harsh climates when distances were negative, but in the opposite directions. PC1 accounted for 0.57of the total variation of PCA for 10 climatic variables (i.e., LAT, Elevation, MAT, MWMT, MAP, DD>5, NFFD, eFFP, MCMT, EMT). The PC1 could be regarded as the growing-season-heat component variable. The significant level of F test for each model was as *p-Value <*0.0001, “***”; <0.001,”**”; <0.01, “*”; <0.05,”`”. Move to cooler (EMT, and DD5) and to the north site (Latitude) will benefit the relative performance.

Table S 7. The scoring protocol of leaf senescence and bud break for aspen trees.

| Score | Bud break stages (Li et al., 2010) |
| --- | --- |
| 0 | Buds are dormant. |
| 1 | Buds are swollen. |
| 2 | Buds are broken. |
| 3 | First new Leave appears (Bud break). |
| 4 | Bud scale are open. |
| 5 | More leaves appeared (leaf out). |
| 6 | Leaved fully unfolded. |
| Score | Leaf senescence stages (Fracheboud et al., 2009) |
| 0 | Leaves are uniformly green. |
| 1 | More dark green leaves than pale green leaves. |
| 2 | More pale green leaves than the dark green. |
| 3 | More green leaves than the yellow. |
| 4 | More yellow leaves than the green leaves. |
| 5 | Only yellow leaves (Leaf coloration). |
| 6 | 80% gold and 20% brown leaves. |
| 7 | Only dark brown leaves with 50% leaf abscission. |
| 8 | More than 90% leaf abscission (bare tree). |

Table S 8. Freezing temperature and time of measurement

| Date | 22-Aug | 12-Sep | 10-Oct |
| --- | --- | --- | --- |
| Test temperature | 8 °C (control),  -5 °C,  -10 °C,  -20 °C,  -30 °C | 8 °C (control), -10 °C,  -30 °C,  -40°C,  -50 °C, and -60 °C | 8 °C (control),  -30 °C,  -60 °C,  -70 °C,  -75°C,  and -80 °C |

Note, 48 trees were sampled on three dates with three different twigs sampled per tree in total.

Table S 9. Heritability ($\hat{h}^{2}$) and Qst of bud break calculated with DoY

| Trait | Score | $\hat{h}^{2}$*** | S.E. | $\hat{Q}_{st}$ | S.E. |
| --- | --- | --- | --- | --- | --- |
| Bud break | 1 | 0.96 | 0.20 | 0.31 | 0.16 |
| DoY | 1.5 | 1.33 | 0.23 | 0.24 | 0.14 |
|  | 2 | 0.93 | 0.19 | 0.23 | 0.13 |
|  | 2.5 | 1.31 | 0.23 | 0.28 | 0.15 |
|  | 3 | 1.21 | 0.22 | 0.23 | 0.13 |
|  | 3.5 | 1.31 | 0.23 | 0.30 | 0.15 |
|  | 4 | 1.25 | 0.23 | 0.24 | 0.13 |
|  | 4.5 | 1.34 | 0.23 | 0.27 | 0.15 |
|  | 5 | 1.31 | 0.23 | 0.15 | 0.10 |

Notes, $\hat{h}^{2}$estimated with half-sib family variances may exceed one.

Method S 10. Artificial freezing measurement procedure:

Tree twigs of eight current year trees from six provenances (48 trees in total) within three eco-regions/provenance groups (Minnesota, central Alberta, and northeast British Columbia) were collected in autumn 2011 on 22-Aug, 12-Sep and 10-Oct at the central Alberta test site (#60). For each testing date we used one twig per freezing temperature from each tree. All twigs from the 48 trees of one testing date were cut into 5 cm pieces and placed in 30 ml high-density polyethylene bottles (Fisherbrand, Fisher Scientific). We added 5 ml of deionized water to the samples prior to freezing treatments to assist ice formation. A programmable freezer (Model 85-3.1A, Scientemp Corp., Adrian, MI, USA) cooled the samples at a rate of approximately 5 °C per hour, and then maintained at the target temperature for one hour before re-warming to 8 °C at which samples were stored for thawing. Then, each segment was cut into five mm pieces, soaked with 20 ml deionized water, stored for 20-24 hours at 8 °C, and manually shaken three times during storage for balancing the electrolyte solution. The electrolyte leakage was measured at room temperature (approximately 20 °C) by using a conductivity meter (Oakton Acorn CON 6 Meter, Oakton Instruments, Vernon Hills, IL, USA). Conductivity readings were recorded before (C­1) and after (C­2) all samples were autoclaved at 100 °C for 50 min.

Method S 11. Prediction of LT50 and LT25

We applied a quadratic regression function estimating the LT50 of each genotype by using PROC MIXED of SAS 9.2 ([SAS Institute Inc. 2008](#_ENREF_45)). The model was in the following form:

$L=b_{k1}(\frac{1}{t_{k}})^{2}+b_{k2}\frac{1}{t_{k}}+b_{k3}D_{k}+b_{k0}+e_{k}$ (10)

where L was the cell lysis (%), $\frac{1}{t_{k}}$ was the inverse of treatment temperature for *k*-th family (k =1 to 6); $D_{k}$ was the sampling date (DoY) of provenance k; e was the residual of *k*-th family of eight twigs (8 per family). $b_{k0}$ to $b_{k3}$were all regression coefficients of the k-th family for this model. When L=0.5, the LT50 of relevant date D was calculated for the family-k by solving the equation. And only square root of the LT solutions within the measurable freezing temperature range ($-80<t_{k}<0$ ) was kept, which was within the range of treatment freezing temperature. Akaike information criterion (AIC) and details of the regression coefficients were presented in Table S 5. To present the curve of LT50 overtime in fall, we solved the function when lysis L=0.5. And this function was solved with L=0.25 for LT25 curve.
